# Supplementary material for: Identification and Validation of a Chromosome 4D Quantitative Trait Locus Hotspot Conferring Heat Tolerance in Common Wheat (Triticum aestivum L.)
Source: Plants (Basel). 2022 Mar 9;11(6):729. doi: 10.3390/plants11060729 (PMC8949852; doi:10.3390/plants11060729)
Supplement: Supplementary file 1 [file plants-11-00729-s001.zip › plants-1630464-supplementary.pdf]

**Supplementary Table S1. Phenotype data and genotype data of RILs**

| Name/Line | 35 SL(cm) | 35 RL(cm) | 35WL(cm) | SLI | RLI | WLI | Marker1scaffold72468_211254 | Marker2scaffold72468_211948 | Marker3scaffold4109_249224 | Marker4scaffold38811_2394108 |
|---|---|---|---|---|---|---|---|---|---|---|
| Synthetic W7984 | 11.47 | 8.73 | 20.30 | -0.11 | 0.17 | 0.01 | GG | AA | CC | AA |
| Opata M85 | 11.57 | 10.33 | 21.80 | -0.37 | 0.00 | -0.21 | AA | CC | TT | CC |
| 1 | 9.63 | 4.73 | 14.36 | -0.08202 | 0.609091 | 0.31619 | AA | CC | TT | CC |
| 2 | 8.07 | 8.47 | 16.54 | 0.42766 | 0.42381 | 0.425694 | AA | CC | CC | AA |
| 3 | 11.83 | 6.1 | 17.93 | 0.330504 | 0.599475 | 0.455015 | AA | CC | TT | CC |
| 4 | 10.57 | 5.73 | 16.3 | 0.336472 | 0.618 | 0.473004 | AA | CC | TT | CC |
| 5 | 7.3 | 5.27 | 12.57 | 0.195149 | 0.111298 | 0.162 | AA | CC | TT | AA |
| 6 | 11.93 | 7.1 | 19.03 | 0.087223 | 0.43336 | 0.256641 | GG | AA | TT | CC |
| 7 | 0.93 | 2.83 | 3.76 | 0.947458 | 0.810449 | 0.884769 | GG | AA | TT | CC |
| 8 | 9 | 5.2 | 14.2 | 0.394755 | 0.659463 | 0.528865 | AA | CC | TT | CC |
| 9 | 1.6 | 2.37 | 3.97 | 0.901051 | 0.858084 | 0.879221 | GG | AA | CC | CC |
| 10 | 1.87 | 3.27 | 5.14 | 0.876159 | 0.785292 | 0.830531 | GG | AA | CC | CC |
| 11 | 3.97 | 4.1 | 8.07 | 0.703731 | 0.646552 | 0.6772 | GG | AA | TT | CC |
| 12 | 15.83 | 10.8 | 26.63 | 0.028834 | 0.261791 | 0.139024 | GG | AA | TT | CC |
| 13 | 0.7 | 0.73 | 1.43 | 0.939655 | 0.942063 | 0.940909 | GG | AA | CC | AA |
| 14 | 3.33 | 2.8 | 6.13 | 0.78884 | 0.798995 | 0.793603 | AA | CC | CC | AA |
| 15 | 0.7 | 2 | 2.7 | 0.945184 | 0.821906 | 0.8875 | GG | AA | TT | CC |
| 16 | 12.73 | 10.03 | 22.76 | 0.30551 | 0.253165 | 0.283375 | GG | AA | CC | AA |
| 17 | 10.6 | 11.77 | 22.37 | 0.298013 | 0.161083 | 0.232063 | GG | AA | CC | AA |
| 18 | 2.8 | 4.5 | 7.3 | 0.823566 | 0.680851 | 0.756423 | GG | AA | TT | CC |
| 19 | 12.77 | 10.37 | 23.14 | -0.0493 | 0.059837 | 0.002586 | AG | CA | TT | CA |
| 20 | 2.4 | 2.47 | 4.87 | 0.819955 | 0.818782 | 0.819362 | N | N | N | N |
| 21 | 10.6 | 7.47 | 18.07 | 0.138211 | 0.438346 | 0.294141 | N | N | N | N |
| 22 | 6.07 | 4.57 | 10.64 | 0.657642 | 0.657164 | 0.657437 | GG | AA | TT | CC |
| 23 | 1.25 | 1.1 | 2.35 | 0.902572 | 0.917293 | 0.910065 | GG | AA | CC | AA |
| 24 | 13.63 | 8.77 | 22.4 | 0.019424 | 0.369065 | 0.194245 | AA | CC | CC | AA |
| 25 | 3.07 | 3.27 | 6.34 | 0.83224 | 0.7471 | 0.79699 | GG | AA | CC | AA |
| 26 | 3.7 | 4.7 | 8.4 | 0.770614 | 0.672017 | 0.724228 | GG | AA | TT | AA |
| 27 | 11.83 | 11.63 | 23.46 | 0.196877 | 0.214189 | 0.205554 | AA | CC | TT | CC |

| | | | | | | | | | | |
|---|---|---|---|---|---|---|---|---|---|---|
| 28 | 6.93 | 5.53 | 12.46 | 0.203448 | 0.438579 | 0.328302 | AA | CC | TT | CC |
| 29 | 5.2 | 5.43 | 10.63 | 0.680982 | 0.675433 | 0.678171 | GG | AA | CC | CC |
| 30 | 12.1 | 8.57 | 20.67 | 0.037391 | 0.440965 | 0.25914 | AA | CC | CC | AA |
| 31 | 4.43 | 4.2 | 8.63 | 0.698023 | 0.638865 | 0.671863 | GG | AA | CC | AA |
| 32 | 9.73 | 9.87 | 19.6 | 0.451213 | 0.316008 | 0.390547 | AA | CC | TT | CC |
| 33 | 10.6 | 10.83 | 21.43 | 0.488664 | 0.282781 | 0.401898 | AA | CC | TT | CC |
| 34 | 7.03 | 9.83 | 16.86 | 0.495334 | 0.389441 | 0.438561 | GG | AA | CC | AA |
| 35 | 3.17 | 5.07 | 8.24 | 0.700095 | 0.543243 | 0.619751 | GG | AA | TT | AA |
| 36 | 7.37 | 8.83 | 16.2 | 0.547853 | 0.427738 | 0.489442 | N | N | N | N |
| 37 | 5.33 | 5.83 | 11.16 | 0.590944 | 0.484071 | 0.541307 | GG | AA | TT | CC |
| 38 | 4.2 | 3.33 | 7.53 | 0.760956 | 0.775 | 0.767377 | GG | AA | TT | CC |
| 39 | 6.67 | 9.57 | 16.24 | 0.661936 | 0.40448 | 0.546369 | AA | CC | CC | AA |
| 40 | 2.4 | 3.67 | 6.07 | 0.779209 | 0.70873 | 0.741372 | GG | AA | CC | AA |
| 41 | 7.37 | 7.97 | 15.34 | 0.514173 | 0.468667 | 0.491548 | GG | AA | CC | AA |
| 42 | 14.53 | 9.33 | 23.86 | 0.101422 | 0.269381 | 0.175536 | AA | CC | CC | AA |
| 43 | 10.97 | 8.73 | 19.7 | 0.431606 | 0.379531 | 0.409649 | GG | AA | TT | CC |
| 44 | 15.63 | 11.27 | 26.9 | 0.120428 | 0.190955 | 0.15142 | AA | CC | TT | CC |
| 45 | 13.73 | 9.93 | 23.66 | 0.282279 | 0.35225 | 0.313407 | AA | CC | TT | CC |
| 46 | 5.45 | 5.1 | 10.55 | 0.586181 | 0.630435 | 0.608825 | GG | AA | CC | AA |
| 47 | 7.5 | 8.77 | 16.27 | 0.567224 | 0.473905 | 0.521471 | GG | AA | CC | CC |
| 48 | 12.37 | 10.9 | 23.27 | 0.213605 | 0.307057 | 0.260331 | GG | AA | TT | CC |
| 49 | 13.73 | 10.37 | 24.1 | 0.281152 | 0.297901 | 0.288456 | AA | CC | TT | CC |
| 50 | 10.27 | 8.93 | 19.2 | 0.235294 | 0.360773 | 0.29927 | N | N | N | N |
| 51 | 13.77 | 10.17 | 23.94 | 0.155215 | 0.259286 | 0.202797 | GG | AA | TT | AA |
| 52 | 10.33 | 6 | 16.33 | 0.388757 | 0.583333 | 0.478275 | GG | AA | TT | CC |
| 53 | 14.07 | 11.3 | 25.37 | 0.122271 | 0.222299 | 0.16983 | AA | CC | CC | CC |
| 54 | 6.63 | 5.9 | 12.53 | 0.546822 | 0.519152 | 0.534201 | AA | CC | TT | CC |
| 55 | 10.07 | 10.03 | 20.1 | 0.151643 | 0.106857 | 0.12987 | N | N | N | N |
| 56 | 13.7 | 8.83 | 22.53 | 0.197422 | 0.38381 | 0.282484 | N | N | N | N |
| 57 | 14.87 | 11.77 | 26.64 | 0.178453 | 0.101527 | 0.146154 | GG | AA | TT | CC |
| 58 | 7.3 | 6.37 | 13.67 | 0.611082 | 0.603609 | 0.607635 | GG | AA | CC | CC |
| 59 | 12.33 | 8.9 | 21.23 | 0.133521 | 0.446173 | 0.29934 | AA | CC | TT | AA |
| 60 | 11.23 | 11.37 | 22.6 | 0.332739 | 0.267869 | 0.301607 | GG | AA | TT | CC |

| | | | | | | | | | | |
|---|---|---|---|---|---|---|---|---|---|---|
| 61 | 5.67 | 5.2 | 10.87 | 0.514139 | 0.577236 | 0.546516 | GG | AA | TT | CC |
| 62 | 8.93 | 4.93 | 13.86 | 0.466228 | 0.650355 | 0.550438 | N | N | N | N |
| 63 | 15.2 | 10.97 | 26.17 | 0.2 | 0.134175 | 0.173666 | AA | CC | TT | CC |
| 64 | 14.93 | 12.03 | 26.96 | 0.182813 | 0.134532 | 0.161952 | AA | CC | CC | AA |
| 65 | 9.93 | 8 | 17.93 | 0.398182 | 0.477124 | 0.436164 | GG | AA | TT | CC |
| 66 | 7.23 | 5.63 | 12.86 | 0.589904 | 0.627892 | 0.607448 | AA | CC | CC | AA |
| 67 | 11.2 | 14.67 | 25.87 | 0.382239 | -0.07315 | 0.186478 | GG | AA | CC | CC |
| 68 | 8.3 | 4.9 | 13.2 | 0.441829 | 0.554545 | 0.489756 | GG | AA | TT | AA |
| 69 | 7.53 | 6.77 | 14.3 | 0.513251 | 0.427726 | 0.47619 | GG | AA | TT | CC |
| 70 | 4.87 | 3.4 | 8.27 | 0.675981 | 0.742424 | 0.707049 | GG | AA | TT | AA |
| 71 | 8.77 | 5.63 | 14.4 | 0.270989 | 0.484904 | 0.372822 | GG | AA | TC | AA |
| 72 | 9.97 | 13.1 | 23.07 | 0.140517 | -0.0023 | 0.064856 | AA | CC | TT | CC |
| 73 | 15.67 | 12.77 | 28.44 | 0.020625 | 0.092395 | 0.054207 | AA | CC | CC | AA |
| 74 | 14.53 | 14.53 | 29.06 | 0.084436 | -0.0305 | 0.030364 | AA | CC | CC | AA |
| 75 | 17.23 | 9.63 | 26.86 | 0.00806 | 0.346232 | 0.16324 | GG | AA | TT | CC |
| 76 | 15.7 | 13.87 | 29.57 | 0.200204 | -0.03276 | 0.105566 | AA | CC | TT | AA |
| 77 | 7.67 | 4.2 | 11.87 | 0.441369 | 0.652893 | 0.540457 | AA | CC | TT | CC |
| 78 | 9.2 | 6.97 | 16.17 | 0.497268 | 0.479851 | 0.489905 | AA | CC | CC | CC |
| 79 | 15.9 | 12.17 | 28.07 | -0.20729 | 0.109729 | -0.04583 | AA | CC | TT | AA |
| 80 | 2.87 | 2.13 | 5 | 0.785821 | 0.801491 | 0.792789 | AA | CC | CC | AA |
| 81 | 7.33 | 3.7 | 11.03 | 0.321296 | 0.646609 | 0.481429 | AA | CC | CC | AA |
| 82 | 8.37 | 6.27 | 14.64 | 0.369254 | 0.443656 | 0.403423 | GG | AA | TT | CC |
| 83 | 13.2 | 8.07 | 21.27 | 0.265442 | 0.36805 | 0.308068 | N | N | N | N |
| 84 | 7.3 | 4.53 | 11.83 | 0.355693 | 0.541033 | 0.441981 | GG | AA | TT | CC |
| 85 | 4.3 | 3.23 | 7.53 | 0.785322 | 0.769286 | 0.778725 | GG | AA | TT | CC |
| 86 | 6.8 | 6.17 | 12.97 | 0.489872 | 0.410697 | 0.455042 | GG | AA | TT | CC |
| 87 | 10.4 | 8.87 | 19.27 | 0.19815 | 0.200901 | 0.199418 | GG | AA | TT | CC |
| 88 | 10.87 | 9.13 | 20 | 0.307643 | 0.179695 | 0.254566 | AA | CC | TT | CC |
| 89 | 5.17 | 4 | 9.17 | 0.610399 | 0.641898 | 0.624795 | GG | AA | CC | AA |
| 90 | 3.83 | 2.57 | 6.4 | 0.707634 | 0.771961 | 0.737382 | N | N | N | N |
| 91 | 13.17 | 11.8 | 24.97 | -0.0621 | -0.06594 | -0.06391 | N | N | N | N |
| 92 | 14.53 | 10.93 | 25.46 | 0.145294 | 0.211968 | 0.175251 | GG | AA | TT | CC |
| 93 | 14.23 | 12.1 | 26.33 | 0.212507 | 0.163787 | 0.190842 | GG | AA | CC | CC |

| | | | | | | | | | | |
|---|---|---|---|---|---|---|---|---|---|---|
| 94 | 11.6 | 12.2 | 23.8 | 0.416205 | 0.034046 | 0.267692 | GG | AA | CC | CC |
| 95 | 14.13 | 12.67 | 26.8 | 0.142077 | -0.08942 | 0.046263 | AA | CC | CC | AA |
| 96 | 9.4 | 5.6 | 15 | 0.397436 | 0.549477 | 0.464859 | GG | AA | CC | AA |
| 97 | 14.8 | 12.43 | 27.23 | 0.130945 | 0.14863 | 0.139108 | AA | CC | CC | AA |
| 98 | 18.7 | 14.17 | 32.87 | 0.026042 | 0.015972 | 0.021726 | AA | CC | TT | AA |
| 99 | 12.87 | 12.37 | 25.24 | 0.215244 | 0.053558 | 0.143536 | GG | AA | CC | AA |
| 100 | 10.3 | 8.03 | 18.33 | 0.046296 | 0.356055 | 0.212291 | AA | CC | CC | AA |
| 101 | 16 | 12.3 | 28.3 | -0.14531 | -0.025 | -0.08972 | GG | AA | CC | CC |
| 102 | 16.75 | 13.97 | 30.72 | -0.30859 | -0.08043 | -0.19394 | N | N | N | N |
| 103 | 9.67 | 8.93 | 18.6 | 0.254433 | 0.127077 | 0.198276 | N | N | N | N |
| 104 | 10.25 | 9.75 | 20 | 0.379915 | 0.268567 | 0.330208 | AA | CC | CC | AA |
| 105 | 1.65 | 2.6 | 4.25 | 0.880866 | 0.761468 | 0.828283 | AA | CC | CC | AA |
| 106 | 12.27 | 10.83 | 23.1 | 0.163599 | 0.110107 | 0.139344 | AA | CC | CC | CC |
| 110 | 12.47 | 8.83 | 21.3 | 0.215723 | 0.325954 | 0.265517 | AA | CC | CC | AA |
| 111 | 10.97 | 10 | 20.97 | 0.10813 | 0.03568 | 0.074989 | GG | AA | TT | CC |
| 112 | 7.17 | 7.53 | 14.7 | 0.352304 | 0.223711 | 0.292248 | AA | CC | CC | AA |
| 113 | 6.05 | 5.9 | 11.95 | 0.412621 | 0.486957 | 0.451835 | N | N | N | N |
| 114 | 11.3 | 7.75 | 19.05 | 0.150376 | 0.326087 | 0.231855 | AA | CC | TT | AA |

N means no data

Synthetic is the susceptible parent and therefore its alleles are considered negative alleles, whereas Opata is the tolerant parent and its alleles are considered positive alleles

**Supplementary Table S2. All 572 genes within the QTL hotspot.**

| Gene-ID | Blast-Hit-Accession | Human-Readable-Description |
|---|---|---|
| TraesCS4D01G005900LC.1 | tr\|A0A0B0M763\|A0A0B0M763_GOSAR | Transport protein sec23 |
| TraesCS4D01G006000LC.1 | AT2G02520.1 | RNA-directed DNA polymerase (reverse transcriptase)-related family protein |
| TraesCS4D01G006100LC.1 | sp\|P30680\|SSR2_RAT | Somatostatin receptor type 2 |
| TraesCS4D01G006200LC.1 | tr\|A0A1D5Y7E4\|A0A1D5Y7E4_WHEAT | 3-ketoacyl-CoA synthase |
| TraesCS4D01G006300LC.1 | tr\|A0A0A9R9I2\|A0A0A9R9I2_ARUDO | HIRA |
| TraesCS4D01G006400LC.1 | tr\|A0A1J3I0I0\|A0A1J3I0I0_NOCCA | Protein ECERIFERUM 3 |
| TraesCS4D01G006500LC.1 | AT4G14170.1 | Pentatricopeptide repeat (PPR) superfamily protein |
| TraesCS4D01G006600LC.1 | AT1G32210.2 | Defender against death (DAD family) protein |
| TraesCS4D01G006600LC.2 | AT1G29750.1 | receptor-like kinase in flowers 1 |
| TraesCS4D01G006600LC.3 | sp\|Q66802\|L_EBOSM | RNA-directed RNA polymerase L |
| TraesCS4D01G006700LC.1 | AT1G33710.1 | RNA-directed DNA polymerase (reverse transcriptase)-related family protein |
| TraesCS4D01G006800LC.1 | sp\|Q5NI06\|LPXD1_FRATT | UDP-3-O-acylglucosamine N-acyltransferase 1 |
| TraesCS4D01G006900LC.1 | sp\|Q87AH7\|SYE_XYLFT | Glutamate--tRNA ligase |
| TraesCS4D01G007000LC.1 | tr\|Q6XW38\|Q6XW38_ARATH | Resistance protein RPP8-like protein |
| TraesCS4D01G007100LC.1 | tr\|Q2QX51\|Q2QX51_ORYSJ | Transposon protein, putative, Pong sub-class |
| TraesCS4D01G007200LC.1 | tr\|A0A0K9Q0G9\|A0A0K9Q0G9_ZOSMR | Dihydrolipoamide acetyltransferase component of pyruvate dehydrogenase complex |
| TraesCS4D01G007300LC.1 | tr\|A0A151S0N2\|A0A151S0N2_CAJCA | Serine/threonine protein phosphatase 7 long form isogeny |
| TraesCS4D01G007400LC.1 | tr\|A0A0B2RA84\|A0A0B2RA84_GLYSO | Cell surface glycoprotein 1 |

| | | |
|---|---|---|
| TraesCS4D01G007500LC.1 | tr\|A0A0K9NSP8\|A0A0K9NSP8_ZOSMR | MYB transcription factor |
| TraesCS4D01G007600LC.1 | AT3G58030.4 | RING/U-box superfamily protein |
| TraesCS4D01G007700LC.1 | sp\|Q44494\|ALGE1_AZOVI | Poly(beta-D-mannuronate) C5 epimerase 1 |
| TraesCS4D01G007800LC.1 | AT5G24155.3 | FAD/NAD(P)-binding oxidoreductase family protein |
| TraesCS4D01G007900LC.1 | sp\|Q80TS8\|SE1L3_MOUSE | Protein sel-1 homolog 3 |
| TraesCS4D01G008000LC.1 | tr\|A0A1J3J004\|A0A1J3J004_NOCCA | Retrovirus-related Pol polyprotein from transposon TNT 1-94 |
| TraesCS4D01G008100LC.1 | sp\|P63465\|ACPS_BRUSU | Holo-[acyl-carrier-protein] synthase |
| TraesCS4D01G008200LC.1 | sp\|Q7CQK1\|MDTJ_SALTY | Spermidine export protein MdtJ |
| TraesCS4D01G008300LC.1 | sp\|Q1R8U4\|MURQ_ECOUT | N-acetylmuramic acid 6-phosphate etherase |
| TraesCS4D01G008400LC.1 | AT2G38100.3 | proton-dependent oligopeptide transport (POT) family protein |
| TraesCS4D01G008500LC.1 | tr\|Q10PF4\|Q10PF4_ORYSJ | Retrotransposon protein, putative, unclassified, expressed |
| TraesCS4D01G008600LC.1 | tr\|A0A0B2R0V5\|A0A0B2R0V5_GLYSO | LINE-1 reverse transcriptase like |
| TraesCS4D01G008700LC.1 | tr\|A0A161V440\|A0A161V440_9MAGN | NAD(P)H-quinone oxidoreductase subunit 2, chloroplastic |
| TraesCS4D01G008800LC.1 | tr\|A2Q1A3\|A2Q1A3_MEDTR | RNA-directed DNA polymerase (Reverse transcriptase) |
| TraesCS4D01G008900LC.1 | | Unknown protein |
| TraesCS4D01G009000LC.1 | tr\|A0A191TDL0\|A0A191TDL0_HORVV | NADH-ubiquinone oxidoreductase chain 4 |
| TraesCS4D01G009100LC.1 | sp\|P49609\|ACON_GRAGA | Aconitate hydratase, mitochondrial |
| TraesCS4D01G009200LC.1 | tr\|E6Y5Q2\|E6Y5Q2_SOLLC | Gag-pol polyprotein |
| TraesCS4D01G009300LC.1 | tr\|Q00RI1\|Q00RI1_ORYSA | tRNA-dihydrouridine synthase |
| TraesCS4D01G009400LC.1 | tr\|A0A059Q1S5\|A0A059Q1S5_9POAL | DNA helicase |

| | | |
|---|---|---|
| TraesCS4D01G009500LC.1 | tr\|Q2R0Z1\|Q2R0Z1_ORYSJ | AT hook motif-containing protein, putative |
| TraesCS4D01G009600LC.1 | AT3G06340.4 | DNAJ heat shock N-terminal domain-containing protein |
| TraesCS4D01G009700LC.1 | tr\|Q5SMW3\|Q5SMW3_ORYSJ | Cyst nematode resistance protein-like |
| TraesCS4D01G009800LC.1 | sp\|A1TDK2\|KGD_MYCVP | Multifunctional 2-oxoglutarate metabolism enzyme |
| TraesCS4D01G009900LC.1 | tr\|A0A1J3HNP1\|A0A1J3HNP1_NOCCA | Glutathione S-transferase T3 |
| TraesCS4D01G010000LC.1 | AT2G02520.1 | RNA-directed DNA polymerase (reverse transcriptase)-related family protein |
| TraesCS4D01G010100LC.1 | tr\|Q2QUR7\|Q2QUR7_ORYSJ | Retrotransposon protein, putative, LINE subclass |
| TraesCS4D01G010200LC.1 | tr\|Q53NJ9\|Q53NJ9_ORYSJ | Retrotransposon protein, putative, unclassified |
| TraesCS4D01G010300LC.1 | sp\|O31971\|YOMM_BACSU | SPBc2 prophage-derived recombinase-like protein YomM |
| TraesCS4D01G010400LC.1 | tr\|W9SBM0\|W9SBM0_9ROSA | E3 ubiquitin-protein ligase SINA-like 2 |
| TraesCS4D01G010500LC.1 | sp\|Q6A7M5\|IF2_PROAC | Translation initiation factor IF-2 |
| TraesCS4D01G010600LC.1 | tr\|A0A072VES6\|A0A072VES6_MEDTR | Endonuclease/exonuclease/phosphatase family protein |
| TraesCS4D01G010700LC.1 | tr\|F4IJA9\|F4IJA9_ARATH | TTF-type zinc finger protein with HAT dimerisation domain |
| TraesCS4D01G010800LC.1 | tr\|Q2QQZ1\|Q2QQZ1_ORYSJ | Retrotransposon protein, putative, Ty3-gypsy subclass |
| TraesCS4D01G010900LC.1 | tr\|Q6ATT7\|Q6ATT7_ORYSJ | Cytochrome P450, putative |
| TraesCS4D01G011000LC.1 | AT1G48090.6 | calcium-dependent lipid-binding family protein |
| TraesCS4D01G011100LC.1 | tr\|A0A151SVR7\|A0A151SVR7_CAJCA | Gag polyprotein |
| TraesCS4D01G011200LC.1 | sp\|Q4A0G2\|COPZ_STAS1 | Copper chaperone CopZ |
| TraesCS4D01G011300LC.1 | tr\|Q2VEY8\|Q2VEY8_PENAM | Transposase |
| TraesCS4D01G011400LC.1 | tr\|Q2QWY8\|Q2QWY8_ORYSJ | Transposon protein, putative, CACTA, En/Spm sub-class |

| | | |
|---|---|---|
| TraesCS4D01G011500LC.1 | tr\|A0A072VES6\|A0A072VES6_MEDTR | Endonuclease/exonuclease/phosphatase family protein |
| TraesCS4D01G011600LC.1 | tr\|A0A199UNR3\|A0A199UNR3_ANACO | LINE-1 retrotransposable element ORF2 protein |
| TraesCS4D01G011700LC.1 | tr\|Q2QZN1\|Q2QZN1_ORYSJ | Retrotransposon protein, putative, unclassified |
| TraesCS4D01G011800LC.1 | sp\|Q9SN43\|CIPKC_ARATH | CBL-interacting serine/threonine-protein kinase 12 |
| TraesCS4D01G011900LC.1 | AT2G04420.1 | Polynucleotidyl transferase, ribonuclease H-like superfamily protein |
| TraesCS4D01G012000LC.1 | tr\|A2Q338\|A2Q338_MEDTR | RNA-directed DNA polymerase (Reverse transcriptase) |
| TraesCS4D01G012100LC.1 | sp\|Q06702\|CDA1_YEAST | Chitin deacetylase 1 |
| TraesCS4D01G012200LC.1 | AT1G35530.4 | DEAD/DEAH box RNA helicase family protein |
| TraesCS4D01G012300LC.1 | tr\|Q2R3P6\|Q2R3P6_ORYSJ | Transposon protein, putative, Mutator sub-class |
| TraesCS4D01G012400LC.1 | tr\|A0A199V9F3\|A0A199V9F3_ANACO | LINE-1 reverse transcriptase |
| TraesCS4D01G012500LC.1 | tr\|A0A0B2R0V5\|A0A0B2R0V5_GLYSO | LINE-1 reverse transcriptase like |
| TraesCS4D01G012600LC.1 | AT2G01280.2 | Cyclin/Brf1-like TBP-binding protein |
| TraesCS4D01G012700LC.1 | tr\|A0A072VES6\|A0A072VES6_MEDTR | Endonuclease/exonuclease/phosphatase family protein |
| TraesCS4D01G012800LC.1 | tr\|Q7G4Q3\|Q7G4Q3_ORYSJ | Retrotransposon protein, putative, unclassified |
| TraesCS4D01G012900LC.1 | tr\|Q2QUC2\|Q2QUC2_ORYSJ | Retrotransposon protein, putative, unclassified |
| TraesCS4D01G013000LC.1 | tr\|A0A1J3FWP0\|A0A1J3FWP0_NOCCA | LINE-1 reverse transcriptase-like protein |
| TraesCS4D01G013100LC.1 | sp\|C0Q4M9\|KEFF_SALPC | Glutathione-regulated potassium-efflux system ancillary protein KefF |
| TraesCS4D01G013200LC.1 | AT2G41590.1 | Ta11-like non-LTR retrotransposon |
| TraesCS4D01G013300LC.1 | AT4G10590.4 | ubiquitin-specific protease 10 |
| TraesCS4D01G013400LC.1 | sp\|P42291\|DRD1C_XENLA | D(1C) dopamine receptor |

| | | |
|---|---|---|
| TraesCS4D01G013500LC.1 | tr\|Q53KL9\|Q53KL9_ORYSJ | Retrotransposon protein, putative, unclassified |
| TraesCS4D01G013600LC.1 | tr\|M8C2B7\|M8C2B7_AEGTA | Retrovirus-related Pol polyprotein from transposon TNT 1-94 |
| TraesCS4D01G013700LC.1 | tr\|B9I5G6\|B9I5G6_POPTR | Ankyrin repeat family protein |
| TraesCS4D01G013800LC.1 | tr\|A0A151RQU3\|A0A151RQU3_CAJCA | Zinc finger MYM-type protein 1 |
| TraesCS4D01G013900LC.1 | AT5G42400.8 | SET domain protein 25 |
| TraesCS4D01G014000LC.1 | AT3G50380.4 | vacuolar protein sorting-associated protein, putative (DUF1162) |
| TraesCS4D01G014100LC.1 | tr\|A0A0B2SAW9\|A0A0B2SAW9_GLYSO | LINE-1 reverse transcriptase like |
| TraesCS4D01G014200LC.1 | tr\|A0A072VD17\|A0A072VD17_MEDTR | Endonuclease/exonuclease/phosphatase family protein |
| TraesCS4D01G014300LC.1 | AT4G30110.2 | heavy metal atpase 2 |
| TraesCS4D01G014400LC.1 | tr\|M8C940\|M8C940_AEGTA | DNA (Cytosine-5)-methyltransferase 1 |
| TraesCS4D01G014400LC.2 | tr\|M8C940\|M8C940_AEGTA | DNA (Cytosine-5)-methyltransferase 1 |
| TraesCS4D01G014500LC.1 | tr\|Q10IA6\|Q10IA6_ORYSJ | Retrotransposon protein, putative, unclassified |
| TraesCS4D01G014600LC.1 | tr\|A0A151QLV5\|A0A151QLV5_CAJCA | Retrovirus-related Pol polyprotein from transposon TNT 1-94 |
| TraesCS4D01G014700LC.1 | tr\|A0A151R424\|A0A151R424_CAJCA | Zinc finger MYM-type protein 1 |
| TraesCS4D01G014800LC.1 | sp\|P96178\|RPOC_WEIPA | DNA-directed RNA polymerase subunit beta' |
| TraesCS4D01G014900LC.1 | AT2G43410.6 | RNA binding protein |
| TraesCS4D01G015000LC.1 | tr\|W6JLH3\|W6JLH3_LILLO | Argonaute protein |
| TraesCS4D01G015100LC.1 | tr\|A0A059Q1J2\|A0A059Q1J2_9POAL | GATA transcription factor |
| TraesCS4D01G015100LC.2 | tr\|A0A059Q1J2\|A0A059Q1J2_9POAL | GATA transcription factor |
| TraesCS4D01G015200LC.1 | sp\|Q0UY20\|ATG1_PHANO | Serine/threonine-protein kinase atg1 |

| | | |
|---|---|---|
| TraesCS4D01G015300LC.1 | tr\|G4WH81\|G4WH81_ARALY | Serine/threonine-protein kinase |
| TraesCS4D01G015400LC.1 | tr\|W5EPD6\|W5EPD6_WHEAT | WAT1-related protein |
| TraesCS4D01G015500LC.1 | tr\|A0A151QY44\|A0A151QY44_CAJCA | Transposon TX1 uncharacterized |
| TraesCS4D01G015600LC.1 | tr\|Q10IA6\|Q10IA6_ORYSJ | Retrotransposon protein, putative, unclassified |
| TraesCS4D01G015700LC.1 | tr\|A0A1D1Y9G7\|A0A1D1Y9G7_9ARAE | 26S protease regulatory subunit 6B |
| TraesCS4D01G015800LC.1 | tr\|Q53MP1\|Q53MP1_ORYSJ | Retrotransposon protein, putative, unclassified |
| TraesCS4D01G015900LC.1 | AT4G14760.4 | kinase interacting (KIP1-like) family protein |
| TraesCS4D01G016000LC.1 | tr\|A0A1D1YMQ7\|A0A1D1YMQ7_9ARAE | NADH-ubiquinone oxidoreductase subunit |
| TraesCS4D01G016100LC.1 | sp\|Q5M1F4\|SSTT_STRT1 | Serine/threonine transporter SstT |
| TraesCS4D01G016200LC.1 | tr\|A0A0B2Q563\|A0A0B2Q563_GLYSO | Non-specific serine/threonine protein kinase |
| TraesCS4D01G016300LC.1 | tr\|A0A1J3HNP1\|A0A1J3HNP1_NOCCA | Glutathione S-transferase T3 |
| TraesCS4D01G016400LC.1 | AT5G62150.1 | peptidoglycan-binding LysM domain-containing protein |
| TraesCS4D01G016500LC.1 | AT1G10000.1 | Ribonuclease H-like superfamily protein |
| TraesCS4D01G016600LC.1 | tr\|Q10MY0\|Q10MY0_ORYSJ | Transposon protein, putative, Mutator sub-class, expressed |
| TraesCS4D01G016700LC.1 | tr\|Q2R1G5\|Q2R1G5_ORYSJ | Transposon protein, putative, Mutator sub-class, expressed |
| TraesCS4D01G016800LC.1 | tr\|Q2R3J1\|Q2R3J1_ORYSJ | Transposon protein, putative, Mutator sub-class |
| TraesCS4D01G016900LC.1 | tr\|A0A151SHP6\|A0A151SHP6_CAJCA | Serine/threonine protein phosphatase 7 long form isogeny |
| TraesCS4D01G017000LC.1 | tr\|S5R947\|S5R947_9ROSA | NAC domain protein |
| TraesCS4D01G017100LC.1 | tr\|Q2QX51\|Q2QX51_ORYSJ | Transposon protein, putative, Pong sub-class |
| TraesCS4D01G017200LC.1 | tr\|A0A151RQB0\|A0A151RQB0_CAJCA | ATP-dependent DNA helicase PIF1 |

| | | |
|---|---|---|
| TraesCS4D01G017300LC.1 | tr\|Q2R0Z1\|Q2R0Z1_ORYSJ | AT hook motif-containing protein, putative |
| TraesCS4D01G017400LC.1 | tr\|Q2R0Z1\|Q2R0Z1_ORYSJ | AT hook motif-containing protein, putative |
| TraesCS4D01G017500LC.1 | tr\|Q9AYF0\|Q9AYF0_ORYSJ | Helicase-like protein |
| TraesCS4D01G017600LC.1 | tr\|A0A0V0IM05\|A0A0V0IM05_SOLCH | Importin subunit alpha |
| TraesCS4D01G017700LC.1 | tr\|Q5JLQ0\|Q5JLQ0_ORYSJ | F-box domain containing protein-like |
| TraesCS4D01G017800LC.1 | tr\|Q2QYQ5\|Q2QYQ5_ORYSJ | Retrotransposon protein, putative, unclassified, expressed |
| TraesCS4D01G017900LC.1 | tr\|A0A151QMR0\|A0A151QMR0_CAJCA | Transposon TX1 uncharacterized |
| TraesCS4D01G018000LC.1 | tr\|A0A072VGJ8\|A0A072VGJ8_MEDTR | Endonuclease/exonuclease/phosphatase family protein |
| TraesCS4D01G018100LC.1 | tr\|A0A151SQ16\|A0A151SQ16_CAJCA | Retrovirus-related Pol polyprotein LINE-1 |
| TraesCS4D01G018200LC.1 | tr\|A0A059Q2Q5\|A0A059Q2Q5_9POAL | DNA-(apurinic or apyrimidinic site) lyase |
| TraesCS4D01G018300LC.1 | tr\|M8BU25\|M8BU25_AEGTA | Protein FAR1-RELATED SEQUENCE 5 |
| TraesCS4D01G018400LC.1 | tr\|A2Q1A3\|A2Q1A3_MEDTR | RNA-directed DNA polymerase (Reverse transcriptase) |
| TraesCS4D01G018500LC.1 | tr\|A0A0A9BDB4\|A0A0A9BDB4_ARUDO | Calcium-dependent protein kinase, isoform 2 |
| TraesCS4D01G018600LC.1 | AT1G27090.1 | glycine-rich protein |
| TraesCS4D01G018700LC.1 | tr\|M8B215\|M8B215_AEGTA | Heat stress transcription factor A-9 |
| TraesCS4D01G018800LC.1 | tr\|B6SWD0\|B6SWD0_MAIZE | Light-mediated development protein DET1 |
| TraesCS4D01G018800LC.2 | sp\|B2AHC1\|SUCC_CUPTR | Succinate--CoA ligase [ADP-forming] subunit beta |
| TraesCS4D01G018900LC.1 | tr\|Q7XE51\|Q7XE51_ORYSJ | Retrotransposon protein, putative, unclassified |
| TraesCS4D01G019000LC.1 | sp\|A4IZJ6\|SYGA_FRATW | Glycine--tRNA ligase alpha subunit |
| TraesCS4D01G019100LC.1 | sp\|B3LY22\|EIF3A_DROAN | Eukaryotic translation initiation factor 3 subunit A |

| | | |
|---|---|---|
| TraesCS4D01G019200LC.1 | tr\|Q6YUC1\|Q6YUC1_ORYSJ | F-box protein family-like |
| TraesCS4D01G019300LC.1 | AT3G24255.3 | RNA-directed DNA polymerase (reverse transcriptase)-related family protein |
| TraesCS4D01G019400LC.1 | sp\|P0C6X3\|R1AB_CVHN2 | Replicase polyprotein 1ab |
| TraesCS4D01G019500LC.1 | | Unknown protein |
| TraesCS4D01G019600LC.1 | sp\|Q8PQ19\|PUR9_XANAC | Bifunctional purine biosynthesis protein PurH |
| TraesCS4D01G019700LC.1 | tr\|Q2QS92\|Q2QS92_ORYSJ | Transposable element protein, putative, Retrotrans_gag |
| TraesCS4D01G019800LC.1 | sp\|B2S334\|NTPA_TREPS | Non-canonical purine NTP pyrophosphatase |
| TraesCS4D01G019900LC.1 | tr\|Q53L77\|Q53L77_ORYSJ | Transposable element protein, putative |
| TraesCS4D01G020000LC.1 | tr\|A0A199UJV5\|A0A199UJV5_ANACO | RING finger and transmembrane domain-containing protein 2 |
| TraesCS4D01G020100LC.1 | tr\|A0A061FVD3\|A0A061FVD3_THECC | BED zinc finger,hAT family dimerization domain |
| TraesCS4D01G020200LC.1 | tr\|A0A061G8Z6\|A0A061G8Z6_THECC | BED zinc finger,hAT family dimerization domain |
| TraesCS4D01G020300LC.1 | tr\|Q109Y5\|Q109Y5_ORYSJ | Retrotransposon protein, putative, Ty3-gypsy subclass |
| TraesCS4D01G020400LC.1 | tr\|Q2QZQ1\|Q2QZQ1_ORYSJ | Retrotransposon protein, putative, Ty3-gypsy subclass |
| TraesCS4D01G020500LC.1 | tr\|Q109Y4\|Q109Y4_ORYSJ | Retrotransposon protein, putative, Ty3-gypsy subclass |
| TraesCS4D01G020600LC.1 | AT5G63570.2 | glutamate-1-semialdehyde-2,1-aminomutase |
| TraesCS4D01G020700LC.1 | tr\|B9HLX2\|B9HLX2_POPTR | Glycine-rich family protein |
| TraesCS4D01G020800LC.1 | tr\|S5R947\|S5R947_9ROSA | NAC domain protein |
| TraesCS4D01G020900LC.1 | tr\|A0A1J3J691\|A0A1J3J691_NOCCA | Retrovirus-related Pol polyprotein from transposon TNT 1-94 |
| TraesCS4D01G021000LC.1 | tr\|D8UEJ6\|D8UEJ6_VOLCA | Ankyrin-repeat protein |
| TraesCS4D01G021100LC.1 | tr\|Q8H2A6\|Q8H2A6_ANACO | Germin-like protein |

| | | |
|---|---|---|
| TraesCS4D01G021200LC.1 | AT3G04030.3 | Homeodomain-like superfamily protein |
| TraesCS4D01G021300LC.1 | AT1G55265.1 | DUF538 family protein, putative (Protein of unknown function, DUF538) |
| TraesCS4D01G021400LC.1 | sp\|Q9SW11\|PUB35_ARATH | U-box domain-containing protein 35 |
| TraesCS4D01G021500LC.1 | tr\|G7LC68\|G7LC68_MEDTR | Ubiquitin carboxyl-terminal hydrolase |
| TraesCS4D01G021600LC.1 | sp\|Q7NY13\|IF2_CHRVO | Translation initiation factor IF-2 |
| TraesCS4D01G021700LC.1 | tr\|A0A0B0MZ94\|A0A0B0MZ94_GOSAR | tRNA (Ile)-lysidine synthase |
| TraesCS4D01G021800LC.1 | AT4G16990.9 | disease resistance protein (TIR-NBS class) |
| TraesCS4D01G021900LC.1 | AT2G45540.6 | WD-40 repeat family protein / beige-like protein |
| TraesCS4D01G022000LC.1 | tr\|A0A0A9JBG3\|A0A0A9JBG3_ARUDO | 50S ribosomal protein L18 |
| TraesCS4D01G022100LC.1 | tr\|Q10M31\|Q10M31_ORYSJ | Transposon protein, putative, Mutator sub-class |
| TraesCS4D01G022200LC.1 | tr\|Q9FH11\|Q9FH11_ARATH | Mutator-like transposase-like |
| TraesCS4D01G022300LC.1 | tr\|Q10M31\|Q10M31_ORYSJ | Transposon protein, putative, Mutator sub-class |
| TraesCS4D01G022400LC.1 | tr\|Q7XDU8\|Q7XDU8_ORYSJ | GRF zinc finger family protein, expressed |
| TraesCS4D01G022500LC.1 | sp\|P21979\|SPAA_STRDO | Cell surface antigen I/II |
| TraesCS4D01G022600LC.1 | tr\|A0A151QXP1\|A0A151QXP1_CAJCA | B3 domain-containing protein family |
| TraesCS4D01G022700LC.1 | tr\|A0A1D1YX54\|A0A1D1YX54_9ARAE | La-related protein |
| TraesCS4D01G022700LC.2 | tr\|Q5NBG8\|Q5NBG8_ORYSJ | La related protein-like |
| TraesCS4D01G022800LC.1 | AT1G03080.3 | kinase interacting (KIP1-like) family protein |
| TraesCS4D01G022900LC.1 | AT4G10613.1 | RNA-directed DNA polymerase (reverse transcriptase)-related family protein |
| TraesCS4D01G023000LC.1 | sp\|P16087\|GAG_FIVPE | Gag polyprotein |

| | | |
|---|---|---|
| TraesCS4D01G023100LC.1 | | Unknown protein |
| TraesCS4D01G023200LC.1 | sp\|Q1A250\|GAG_SIVEK | Gag polyprotein |
| TraesCS4D01G023300LC.1 | AT4G33240.7 | 1-phosphatidylinositol-3-phosphate 5-kinase FAB1A |
| TraesCS4D01G023400LC.1 | tr\|I3NM41\|I3NM41_WHEAT | Oleosin |
| TraesCS4D01G023500LC.1 | tr\|A0A1D1YR54\|A0A1D1YR54_9ARAE | Retrovirus-related Pol polyprotein from transposon TNT 1-94 |
| TraesCS4D01G023600LC.1 | sp\|Q8CQ56\|ADH_STAES | Alcohol dehydrogenase |
| TraesCS4D01G023700LC.1 | AT2G03150.2 | ATP/GTP-binding protein family |
| TraesCS4D01G023800LC.1 | sp\|Q8BND5\|QSOX1_MOUSE | Sulfhydryl oxidase 1 |
| TraesCS4D01G023900LC.1 | tr\|A0A151SUT9\|A0A151SUT9_CAJCA | Serine/threonine protein phosphatase 7 long form isogeny |
| TraesCS4D01G024000LC.1 | tr\|A0A0B2RSH3\|A0A0B2RSH3_GLYSO | 3-epi-6-deoxocathasterone 23-monooxygenase |
| TraesCS4D01G024100LC.1 | sp\|Q63TM6\|SYFA_BURPS | Phenylalanine--tRNA ligase alpha subunit |
| TraesCS4D01G024200LC.1 | tr\|A0A072TKF9\|A0A072TKF9_MEDTR | Ycf15 protein, putative |
| TraesCS4D01G024300LC.1 | sp\|O83971\|THYX_TREPA | Flavin-dependent thymidylate synthase |
| TraesCS4D01G024400LC.1 | sp\|Q5P2L1\|COAX_AROAE | Type III pantothenate kinase |
| TraesCS4D01G024500LC.1 | AT2G41350.1 | HAUS augmin-like complex subunit |
| TraesCS4D01G024600LC.1 | tr\|Q2QSZ7\|Q2QSZ7_ORYSJ | Retrotransposon protein, putative, Ty3-gypsy subclass |
| TraesCS4D01G024700LC.1 | tr\|Q2VEY8\|Q2VEY8_PENAM | Transposase |
| TraesCS4D01G024800LC.1 | AT5G55980.1 | serine-rich protein-like protein |
| TraesCS4D01G024900LC.1 | sp\|B5FBM2\|ASTD_VIBFM | N-succinylglutamate 5-semialdehyde dehydrogenase |
| TraesCS4D01G025000LC.1 | tr\|A0A059Q1J1\|A0A059Q1J1_9POAL | Ralf-like protein |

| | | |
|---|---|---|
| TraesCS4D01G025100LC.1 | AT1G12620.1 | Pentatricopeptide repeat (PPR) superfamily protein |
| TraesCS4D01G025200LC.1 | tr\|B9SBS8\|B9SBS8_RICCO | Cysteine protease, putative |
| TraesCS4D01G025300LC.1 | tr\|Q10D55\|Q10D55_ORYSJ | Retrotransposon protein, putative, unclassified |
| TraesCS4D01G025400LC.1 | AT3G24255.5 | RNA-directed DNA polymerase (reverse transcriptase)-related family protein |
| TraesCS4D01G025500LC.1 | sp\|B5FBM2\|ASTD_VIBFM | N-succinylglutamate 5-semialdehyde dehydrogenase |
| TraesCS4D01G025600LC.1 | tr\|M8BMY5\|M8BMY5_AEGTA | Palmitoyl-protein thioesterase 1 |
| TraesCS4D01G025700LC.1 | sp\|Q8FW07\|UGPC_BRUSU | sn-glycerol-3-phosphate import ATP-binding protein UgpC |
| TraesCS4D01G025800LC.1 | sp\|Q9Z1R2\|BAG6_MOUSE | Large proline-rich protein BAG6 |
| TraesCS4D01G025800LC.2 | sp\|P0A125\|RSMG_PSEPU | Ribosomal RNA small subunit methyltransferase G |
| TraesCS4D01G025900LC.1 | | Unknown protein |
| TraesCS4D01G026000LC.1 | tr\|Q0GJY9\|Q0GJY9_9POAL | Transposase |
| TraesCS4D01G026100LC.1 | AT3G19990.2 | E3 ubiquitin-protein ligase |
| TraesCS4D01G026200LC.1 | AT1G23180.2 | ARM repeat superfamily protein |
| TraesCS4D01G026300LC.1 | tr\|Q53NE2\|Q53NE2_ORYSJ | Retrotransposon protein, putative, unclassified |
| TraesCS4D01G026400LC.1 | tr\|M8C2B7\|M8C2B7_AEGTA | Retrovirus-related Pol polyprotein from transposon TNT 1-94 |
| TraesCS4D01G026500LC.1 | sp\|Q89928\|POL_HV2EH | Gag-Pol polyprotein |
| TraesCS4D01G026600LC.1 | tr\|A0A151RAE6\|A0A151RAE6_CAJCA | Transposon Ty3-G Gag-Pol polyprotein |
| TraesCS4D01G026700LC.1 | tr\|M8AUX6\|M8AUX6_TRIUA | ATP synthase subunit alpha |
| TraesCS4D01G026800LC.1 | AT3G12640.3 | RNA binding (RRM/RBD/RNP motifs) family protein |
| TraesCS4D01G026900LC.1 | tr\|Q2RB42\|Q2RB42_ORYSJ | Transposon protein, putative, Mutator sub-class |

| | | |
|---|---|---|
| TraesCS4D01G027000LC.1 | tr\|Q2R0C2\|Q2R0C2_ORYSJ | Transposon protein, putative, Mutator sub-class |
| TraesCS4D01G027000LC.2 | tr\|Q2QXK8\|Q2QXK8_ORYSJ | Transposon protein, putative, Mutator sub-class |
| TraesCS4D01G027000LC.3 | tr\|Q2R0C2\|Q2R0C2_ORYSJ | Transposon protein, putative, Mutator sub-class |
| TraesCS4D01G027000LC.4 | tr\|Q2QXK8\|Q2QXK8_ORYSJ | Transposon protein, putative, Mutator sub-class |
| TraesCS4D01G027100LC.1 | AT1G79950.7 | RAD3-like DNA-binding helicase protein |
| TraesCS4D01G027200LC.1 | sp\|Q9QYF3\|MYO5A_RAT | Unconventional myosin-Va |
| TraesCS4D01G027300LC.1 | tr\|D0U5T0\|D0U5T0_9LAMI | NADH dehydrogenase subunit F |
| TraesCS4D01G027400LC.1 | tr\|M8CJZ3\|M8CJZ3_AEGTA | Protein FAR1-RELATED SEQUENCE 5 |
| TraesCS4D01G027500LC.1 | tr\|M8CJZ3\|M8CJZ3_AEGTA | Protein FAR1-RELATED SEQUENCE 5 |
| TraesCS4D01G027600LC.1 | sp\|Q5HAJ4\|RS6_EHRRW | 30S ribosomal protein S6 |
| TraesCS4D01G027700LC.1 | sp\|Q8CSV3\|RECG_STAES | ATP-dependent DNA helicase RecG |
| TraesCS4D01G027800LC.1 | AT3G58930.5 | F-box/RNI-like superfamily protein |
| TraesCS4D01G027900LC.1 | tr\|A0A072UK42\|A0A072UK42_MEDTR | No-apical-meristem-associated carboxy-terminal domain protein |
| TraesCS4D01G028000LC.1 | tr\|B9GG09\|B9GG09_POPTR | Serine-rich family protein |
| TraesCS4D01G028100LC.1 | AT4G09660.1 | zinc finger MYM-type-like protein |
| TraesCS4D01G028200LC.1 | tr\|A0A151THK7\|A0A151THK7_CAJCA | Retrovirus-related Pol polyprotein from transposon TNT 1-94 |
| TraesCS4D01G028300LC.1 | AT3G25490.1 | Protein kinase family protein |
| TraesCS4D01G028400LC.1 | tr\|Q6H7K3\|Q6H7K3_ORYSJ | Wall-associated kinase-like |
| TraesCS4D01G028500LC.1 | sp\|O64032\|SUNI_BPSPB | Sublancin immunity protein sunI |
| TraesCS4D01G028600LC.1 | AT3G04580.2 | Signal transduction histidine kinase, hybrid-type, ethylene sensor |

| | | |
|---|---|---|
| TraesCS4D01G028700LC.1 | sp\|E9Q784\|ZC3HD_MOUSE | Zinc finger CCCH domain-containing protein 13 |
| TraesCS4D01G028800LC.1 | tr\|W5IF96\|W5IF96_9ROSI | Cytochrome b6-f complex subunit 8 |
| TraesCS4D01G028900LC.1 | AT1G08060.3 | ATP-dependent helicase family protein |
| TraesCS4D01G029000LC.1 | tr\|A0A0U1WZI1\|A0A0U1WZI1_9ORYZ | DNA-directed RNA polymerase subunit beta |
| TraesCS4D01G029100LC.1 | tr\|A0A0M5I8Y8\|A0A0M5I8Y8_ALLSE | DNA-directed RNA polymerase subunit beta |
| TraesCS4D01G029200LC.1 | tr\|R7WAX4\|R7WAX4_AEGTA | DNA-directed RNA polymerase subunit beta |
| TraesCS4D01G029300LC.1 | tr\|K4PMN2\|K4PMN2_FESPR | DNA-directed RNA polymerase subunit beta |
| TraesCS4D01G029400LC.1 | AT5G56640.2 | myo-inositol oxygenase 5 |
| TraesCS4D01G029500LC.1 | tr\|E9KJ86\|E9KJ86_9ORYZ | DNA-directed RNA polymerase subunit |
| TraesCS4D01G029600LC.1 | tr\|M0VEC4\|M0VEC4_HORVV | DNA-directed RNA polymerase subunit |
| TraesCS4D01G029700LC.1 | tr\|A0A193DAC0\|A0A193DAC0_9POAL | DNA-directed RNA polymerase subunit |
| TraesCS4D01G029800LC.1 | tr\|A0A090AKN1\|A0A090AKN1_TRITI | DNA-directed RNA polymerase subunit beta'' |
| TraesCS4D01G029900LC.1 | tr\|A0A142G8K5\|A0A142G8K5_AEGTA | DNA-directed RNA polymerase subunit beta'' |
| TraesCS4D01G030000LC.1 | tr\|A0A142G8K5\|A0A142G8K5_AEGTA | DNA-directed RNA polymerase subunit beta'' |
| TraesCS4D01G030100LC.1 | tr\|A0A090AKN1\|A0A090AKN1_TRITI | DNA-directed RNA polymerase subunit beta'' |
| TraesCS4D01G030200LC.1 | sp\|Q8P5Z4\|PGK_XANCP | Phosphoglycerate kinase |
| TraesCS4D01G030300LC.1 | sp\|B1WQR5\|RL22_CYAA5 | 50S ribosomal protein L22 |
| TraesCS4D01G030400LC.1 | tr\|M7ZKL2\|M7ZKL2_TRIUA | ATP synthase subunit a, chloroplastic |
| TraesCS4D01G030500LC.1 | AT1G18900.4 | Pentatricopeptide repeat (PPR) superfamily protein |
| TraesCS4D01G030600LC.1 | tr\|M8AUX6\|M8AUX6_TRIUA | ATP synthase subunit alpha |

| | | |
|---|---|---|
| TraesCS4D01G030700LC.1 | tr\|A0A1B1W5B5\|A0A1B1W5B5_ECLPR | Photosystem I P700 chlorophyll a apoprotein A2 |
| TraesCS4D01G030800LC.1 | tr\|A0A1B1MSE8\|A0A1B1MSE8_9APIA | Photosystem I P700 chlorophyll a apoprotein A2 |
| TraesCS4D01G030900LC.1 | tr\|S4Z1X0\|S4Z1X0_HORVV | Photosystem I P700 chlorophyll a apoprotein A1 |
| TraesCS4D01G031000LC.1 | tr\|L7NPH8\|L7NPH8_MAGGA | Photosystem I P700 chlorophyll a apoprotein A1 |
| TraesCS4D01G031100LC.1 | tr\|A0A0M4B318\|A0A0M4B318_9ARAE | Photosystem I reaction center subunit IX |
| TraesCS4D01G031200LC.1 | tr\|A0A077RRC9\|A0A077RRC9_WHEAT | 50S ribosomal protein L20 |
| TraesCS4D01G031300LC.1 | sp\|Q0BYA9\|RS12_HYPNA | 30S ribosomal protein S12 |
| TraesCS4D01G031400LC.1 | tr\|A0A0U2KUN5\|A0A0U2KUN5_9POAL | ATP-dependent Clp protease proteolytic subunit |
| TraesCS4D01G031400LC.2 | tr\|A0A126KBF6\|A0A126KBF6_9POAL | ATP-dependent Clp protease proteolytic subunit |
| TraesCS4D01G031500LC.1 | tr\|A0A075VZ19\|A0A075VZ19_AEGSP | Photosystem II CP47 reaction center protein |
| TraesCS4D01G031600LC.1 | tr\|A0A0F6NNB0\|A0A0F6NNB0_9POAL | Photosystem II CP47 reaction center protein |
| TraesCS4D01G031700LC.1 | tr\|A0A075BCK1\|A0A075BCK1_9POAL | Photosystem II CP47 reaction center protein |
| TraesCS4D01G031800LC.1 | tr\|A0A0Q3GQP6\|A0A0Q3GQP6_BRADI | Photosystem II CP47 reaction center protein |
| TraesCS4D01G031900LC.1 | sp\|Q5R8S4\|ERLEC_PONAB | Endoplasmic reticulum lectin 1 |
| TraesCS4D01G032000LC.1 | tr\|U5TXC9\|U5TXC9_SETIT | Cytochrome b6 |
| TraesCS4D01G032100LC.1 | tr\|A0A072TJJ2\|A0A072TJJ2_MEDTR | DNA-directed RNA polymerase subunit alpha |
| TraesCS4D01G032200LC.1 | tr\|A0A0H3V7R1\|A0A0H3V7R1_9ORYZ | Ribosomal protein L36 |
| TraesCS4D01G032300LC.1 | tr\|W5ZNJ6\|W5ZNJ6_DESAN | Translation initiation factor IF-1 |
| TraesCS4D01G032400LC.1 | sp\|Q110B7\|RL14_TRIEI | 50S ribosomal protein L14 |
| TraesCS4D01G032500LC.1 | tr\|A0A072TKH5\|A0A072TKH5_MEDTR | 50S ribosomal protein L16 |

| | | |
|---|---|---|
| TraesCS4D01G032600LC.1 | tr\|S4Z3B2\|S4Z3B2_WHEAT | 30S ribosomal protein S3, chloroplastic |
| TraesCS4D01G032700LC.1 | tr\|S4Z8B0\|S4Z8B0_HORVV | 30S ribosomal protein S3, chloroplastic |
| TraesCS4D01G032800LC.1 | sp\|Q8YPI4\|RL22_NOSS1 | 50S ribosomal protein L22 |
| TraesCS4D01G032900LC.1 | tr\|G0YEB2\|G0YEB2_SARHE | Protein Ycf2 |
| TraesCS4D01G033000LC.1 | tr\|A0A090ARM8\|A0A090ARM8_TRITI | Ycf2 protein |
| TraesCS4D01G033100LC.1 | tr\|A0A078FY29\|A0A078FY29_BRANA | BnaA06g35060D protein |
| TraesCS4D01G033200LC.1 | tr\|A0A1D8KWP2\|A0A1D8KWP2_9POAL | NAD(P)H-quinone oxidoreductase subunit 2, chloroplastic |
| TraesCS4D01G033300LC.1 | tr\|B9SUC9\|B9SUC9_RICCO | Receptor-kinase, putative |
| TraesCS4D01G033400LC.1 | AT1G30590.3 | RNA polymerase I specific transcription initiation factor RRN3 protein |
| TraesCS4D01G033500LC.1 | tr\|Q7G4Q3\|Q7G4Q3_ORYSJ | Retrotransposon protein, putative, unclassified |
| TraesCS4D01G033600LC.1 | AT2G13980.1 | Polynucleotidyl transferase, ribonuclease H-like superfamily protein |
| TraesCS4D01G033700LC.1 | | Unknown protein |
| TraesCS4D01G033800LC.1 | sp\|Q7RTP6\|MICA3_HUMAN | [F-actin]-methionine sulfoxide oxidase MICAL3 |
| TraesCS4D01G033900LC.1 | tr\|A0A072TSI0\|A0A072TSI0_MEDTR | Myb/SANT-like DNA-binding domain protein |
| TraesCS4D01G034000LC.1 | tr\|G7ZV85\|G7ZV85_MEDTR | FAR1-related sequence protein, putative |
| TraesCS4D01G034000LC.2 | sp\|C5BW62\|MRAY_BEUC1 | Phospho-N-acetylmuramoyl-pentapeptide-transferase |
| TraesCS4D01G034100LC.1 | tr\|M8CG74\|M8CG74_AEGTA | 50S ribosomal protein L18 |
| TraesCS4D01G034200LC.1 | tr\|M8D7W2\|M8D7W2_AEGTA | Exonuclease 3'-5' domain-containing protein 1 |
| TraesCS4D01G034300LC.1 | tr\|A0A1J3IDE7\|A0A1J3IDE7_NOCCA | Serine/threonine-protein phosphatase 7 long form-like protein |
| TraesCS4D01G034400LC.1 | AT5G16120.4 | alpha/beta-Hydrolases superfamily protein |

| | | |
|---|---|---|
| TraesCS4D01G034500LC.1 | tr\|A0A0B2P0Q4\|A0A0B2P0Q4_GLYSO | Ubiquitin-like-specific protease ESD4 |
| TraesCS4D01G034500LC.2 | tr\|A0A151STM7\|A0A151STM7_CAJCA | Ubiquitin-like-specific protease 1 |
| TraesCS4D01G034600LC.1 | tr\|M8BH66\|M8BH66_AEGTA | Protein FAR1-RELATED SEQUENCE 5 |
| TraesCS4D01G034700LC.1 | AT3G13690.3 | kinase with adenine nucleotide alpha hydrolases-like domain-containing protein |
| TraesCS4D01G034800LC.1 | tr\|B9ICZ7\|B9ICZ7_POPTR | Gibberellin 20-oxidase family protein |
| TraesCS4D01G034900LC.1 | tr\|A0A151R8A5\|A0A151R8A5_CAJCA | Transposon Ty3-G Gag-Pol polyprotein |
| TraesCS4D01G035000LC.1 | tr\|J3SDF5\|J3SDF5_BETVU | Ty3/gypsy retrotransposon protein |
| TraesCS4D01G035100LC.1 | tr\|A0A151RSW7\|A0A151RSW7_CAJCA | Transposon Ty3-G Gag-Pol polyprotein |
| TraesCS4D01G035200LC.1 | tr\|Q2HS45\|Q2HS45_MEDTR | RNA-directed DNA polymerase (Reverse transcriptase) |
| TraesCS4D01G035300LC.1 | tr\|M8A244\|M8A244_TRIUA | Methylthioribose kinase 1 |
| TraesCS4D01G035400LC.1 | tr\|A0A0B2R0Q2\|A0A0B2R0Q2_GLYSO | LINE-1 reverse transcriptase like |
| TraesCS4D01G035500LC.1 | sp\|O19137\|CPSF4_BOVIN | Cleavage and polyadenylation specificity factor subunit 4 |
| TraesCS4D01G035600LC.1 | AT1G74040.2 | 2-isopropylmalate synthase 1 |
| TraesCS4D01G035700LC.1 | AT3G61680.3 | alpha/beta-Hydrolases superfamily protein |
| TraesCS4D01G035800LC.1 | tr\|M8AVB1\|M8AVB1_AEGTA | Exportin-1 |
| TraesCS4D01G035900LC.1 | tr\|B9GG09\|B9GG09_POPTR | Serine-rich family protein |
| TraesCS4D01G036000LC.1 | tr\|W9R2A9\|W9R2A9_9ROSA | Signal recognition particle 54 kDa protein |
| TraesCS4D01G036100LC.1 | AT3G09510.1 | Ribonuclease H-like superfamily protein |
| TraesCS4D01G036200LC.1 | AT4G09660.1 | zinc finger MYM-type-like protein |
| TraesCS4D01G036300LC.1 | AT4G04650.1 | RNA-directed DNA polymerase (reverse transcriptase)-related family protein |

| | | |
|---|---|---|
| TraesCS4D01G036400LC.1 | tr\|Q9SKD4\|Q9SKD4_ARATH | Polynucleotidyl transferase, ribonuclease H-like superfamily protein |
| TraesCS4D01G036500LC.1 | tr\|A2Q338\|A2Q338_MEDTR | RNA-directed DNA polymerase (Reverse transcriptase) |
| TraesCS4D01G036600LC.1 | tr\|A0A144LHE8\|A0A144LHE8_9BRYO | Dehydration-responsive element-binding protein 5-8 |
| TraesCS4D01G036700LC.1 | AT5G63650.1 | SNF1-related protein kinase 2.5 |
| TraesCS4D01G036800LC.1 | tr\|M7ZCC7\|M7ZCC7_TRIUA | E3 ubiquitin-protein ligase UBR2 |
| TraesCS4D01G036900LC.1 | tr\|A0A0H3V7R1\|A0A0H3V7R1_9ORYZ | Ribosomal protein L36 |
| TraesCS4D01G037000LC.1 | AT2G02520.1 | RNA-directed DNA polymerase (reverse transcriptase)-related family protein |
| TraesCS4D01G037100LC.1 | tr\|A0A199V9F3\|A0A199V9F3_ANACO | LINE-1 reverse transcriptase |
| TraesCS4D01G037200LC.1 | tr\|B9H7F4\|B9H7F4_POPTR | BTB/POZ domain-containing family protein |
| TraesCS4D01G037300LC.1 | tr\|M8AN05\|M8AN05_TRIUA | LRR receptor-like serine/threonine-protein kinase EFR |
| TraesCS4D01G037400LC.1 | tr\|A0A072VES6\|A0A072VES6_MEDTR | Endonuclease/exonuclease/phosphatase family protein |
| TraesCS4D01G037500LC.1 | tr\|A0A0K9PGX3\|A0A0K9PGX3_ZOSMR | DNA-(Apurinic or apyrimidinic site) lyase |
| TraesCS4D01G037600LC.1 | tr\|A0A1J3JXT5\|A0A1J3JXT5_NOCCA | Glutathione S-transferase T3 |
| TraesCS4D01G037700LC.1 | AT1G76170.7 | 2-thiocytidine tRNA biosynthesis protein, TtcA |
| TraesCS4D01G037800LC.1 | tr\|M8BGQ5\|M8BGQ5_AEGTA | Protein FAR1-RELATED SEQUENCE 3 |
| TraesCS4D01G037900LC.1 | tr\|A0A059Q135\|A0A059Q135_9POAL | Pentatricopeptide repeat-containing protein |
| TraesCS4D01G038000LC.1 | sp\|B1KU74\|FABH_CLOBM | 3-oxoacyl-[acyl-carrier-protein] synthase 3 |
| TraesCS4D01G038100LC.1 | tr\|A0A072VES6\|A0A072VES6_MEDTR | Endonuclease/exonuclease/phosphatase family protein |
| TraesCS4D01G038200LC.1 | tr\|Q10LP8\|Q10LP8_ORYSJ | Retrotransposon protein, putative, unclassified |
| TraesCS4D01G038300LC.1 | AT2G26170.2 | cytochrome P450, family 711, subfamily A, polypeptide 1 |

| | | |
|---|---|---|
| TraesCS4D01G038400LC.1 | sp\|P66849\|SSB_NEIMB | Single-stranded DNA-binding protein |
| TraesCS4D01G038500LC.1 | sp\|Q6D9D2\|ARGR_PECAS | Arginine repressor |
| TraesCS4D01G038600LC.1 | tr\|A0A151RPT4\|A0A151RPT4_CAJCA | Retrovirus-related Pol polyprotein from transposon TNT 1-94 |
| TraesCS4D01G038700LC.1 | tr\|A0A151TPU3\|A0A151TPU3_CAJCA | Retrovirus-related Pol polyprotein from transposon TNT 1-94 |
| TraesCS4D01G038800LC.1 | AT4G25440.3 | zinc finger WD40 repeat protein 1 |
| TraesCS4D01G038900LC.1 | tr\|Q10LD3\|Q10LD3_ORYSJ | Retrotransposon protein, putative, Ty3-gypsy subclass |
| TraesCS4D01G039000LC.1 | AT1G60060.2 | Serine/threonine-protein kinase WNK (With No Lysine)-like protein |
| TraesCS4D01G039100LC.1 | AT3G48330.4 | protein-l-isoaspartate methyltransferase 1 |
| TraesCS4D01G039200LC.1 | AT5G65500.2 | U-box domain-containing protein kinase family protein |
| TraesCS4D01G039300LC.1 | AT1G10000.1 | Ribonuclease H-like superfamily protein |
| TraesCS4D01G039400LC.1 | tr\|Q2QSK7\|Q2QSK7_ORYSJ | Retrotransposon protein, putative, LINE subclass |
| TraesCS4D01G039500LC.1 | sp\|Q5EA76\|VWA9_BOVIN | von Willebrand factor A domain-containing protein 9 |
| TraesCS4D01G039600LC.1 | tr\|A0A072URD1\|A0A072URD1_MEDTR | GATA transcription factor-like protein |
| TraesCS4D01G039700LC.1 | sp\|Q5WVN2\|MSBA_LEGPL | Lipid A export ATP-binding/permease protein MsbA |
| TraesCS4D01G039800LC.1 | tr\|A0A1E5VD34\|A0A1E5VD34_9POAL | Mediator of RNA polymerase II transcription subunit 4 |
| TraesCS4D01G039900LC.1 | tr\|A0A1D6AP76\|A0A1D6AP76_WHEAT | Peptidyl-prolyl cis-trans isomerase |
| TraesCS4D01G040000LC.1 | AT1G80270.5 | PENTATRICOPEPTIDE REPEAT 596 |
| TraesCS4D01G040100LC.1 | sp\|P0C220\|SNX2_MACFA | Sorting nexin-2 |
| TraesCS4D01G040200LC.1 | tr\|A0A151SA71\|A0A151SA71_CAJCA | Protein FAR1-RELATED SEQUENCE 5 |
| TraesCS4D01G040300LC.1 | tr\|Q2R446\|Q2R446_ORYSJ | Retrotransposon protein, putative, unclassified |

| | | |
|---|---|---|
| TraesCS4D01G040400LC.1 | tr\|A0A0K9PWT3\|A0A0K9PWT3_ZOSMR | Protein kinase |
| TraesCS4D01G040500LC.1 | tr\|A0A151TAT3\|A0A151TAT3_CAJCA | Retrovirus-related Pol polyprotein from transposon TNT 1-94 |
| TraesCS4D01G040600LC.1 | sp\|O54889\|RPA1_RAT | DNA-directed RNA polymerase I subunit RPA1 |
| TraesCS4D01G040700LC.1 | tr\|A0A1J3HNP1\|A0A1J3HNP1_NOCCA | Glutathione S-transferase T3 |
| TraesCS4D01G040800LC.1 | tr\|Q60DK1\|Q60DK1_ORYSJ | Transposon protein, putative, CACTA, En/Spm sub-class |
| TraesCS4D01G040900LC.1 | tr\|Q2RB42\|Q2RB42_ORYSJ | Transposon protein, putative, Mutator sub-class |
| TraesCS4D01G041000LC.1 | sp\|Q9Z1B3\|PLCB1_MOUSE | 1-phosphatidylinositol 4,5-bisphosphate phosphodiesterase beta-1 |
| TraesCS4D01G041100LC.1 | tr\|A0A061EBG3\|A0A061EBG3_THECC | Ribonuclease H-like superfamily protein |
| TraesCS4D01G041200LC.1 | tr\|G7J9T6\|G7J9T6_MEDTR | Transmembrane protein, putative |
| TraesCS4D01G041300LC.1 | tr\|A0A1J3J691\|A0A1J3J691_NOCCA | Retrovirus-related Pol polyprotein from transposon TNT 1-94 |
| TraesCS4D01G041400LC.1 | tr\|Q2QP27\|Q2QP27_ORYSJ | Transposon protein, putative, Pong sub-class |
| TraesCS4D01G041500LC.1 | sp\|Q9HR49\|PURL_HALSA | Phosphoribosylformylglycinamidine synthase subunit PurL |
| TraesCS4D01G041600LC.1 | tr\|Q53KF9\|Q53KF9_ORYSJ | Transposon protein, putative, CACTA, En/Spm sub-class |
| TraesCS4D01G041700LC.1 | sp\|Q8AAP9\|CYSN_BACTN | Sulfate adenylyltransferase subunit 1 |
| TraesCS4D01G041800LC.1 | tr\|Q9FEI4\|Q9FEI4_9POAL | Receptor-like kinase extracellular domain LRKA14A3 |
| TraesCS4D01G041900LC.1 | sp\|C1D0Z0\|PDXH_DEIDV | Pyridoxine/pyridoxamine 5'-phosphate oxidase |
| TraesCS4D01G042000LC.1 | tr\|B6TCG7\|B6TCG7_MAIZE | Transposon protein Pong sub-class |
| TraesCS4D01G042100LC.1 | AT3G25270.1 | Ribonuclease H-like superfamily protein |
| TraesCS4D01G042200LC.1 | tr\|M5X554\|M5X554_PRUPE | 3-ketoacyl-CoA synthase |
| TraesCS4D01G042300LC.1 | tr\|Q2QNF1\|Q2QNF1_ORYSJ | Retrotransposon protein, putative, unclassified |

| | | |
|---|---|---|
| TraesCS4D01G042400LC.1 | tr\|Q10LY2\|Q10LY2_ORYSJ | Ulp1 protease family, C-terminal catalytic domain containing protein, expressed |
| TraesCS4D01G042500LC.1 | sp\|B3Q5Z7\|AROA_RHOPT | 3-phosphoshikimate 1-carboxyvinyltransferase |
| TraesCS4D01G042600LC.1 | tr\|M8CJZ3\|M8CJZ3_AEGTA | Protein FAR1-RELATED SEQUENCE 5 |
| TraesCS4D01G042700LC.1 | tr\|V4ST04\|V4ST04_9ROSI | Methionine aminopeptidase |
| TraesCS4D01G042800LC.1 | tr\|O80781\|O80781_ARATH | Polynucleotidyl transferase, ribonuclease H-like superfamily protein |
| TraesCS4D01G042900LC.1 | tr\|E5RPJ4\|E5RPJ4_NICBE | Malic enzyme |
| TraesCS4D01G042900LC.2 | sp\|P80691\|LSPI_CARPA | Latex serine proteinase inhibitor |
| TraesCS4D01G043000LC.1 | AT2G21390.1 | Coatomer, alpha subunit |
| TraesCS4D01G043000LC.2 | AT2G21390.1 | Coatomer, alpha subunit |
| TraesCS4D01G043100LC.1 | tr\|A0A0D3EQN3\|A0A0D3EQN3_9ORYZ | Serine/threonine-protein kinase |
| TraesCS4D01G043200LC.1 | tr\|Q37681\|Q37681_WHEAT | ATP synthase subunit 9, mitochondrial |
| TraesCS4D01G043300LC.1 | AT2G34320.1 | Polynucleotidyl transferase, ribonuclease H-like superfamily protein |
| TraesCS4D01G043400LC.1 | tr\|A2Q338\|A2Q338_MEDTR | RNA-directed DNA polymerase (Reverse transcriptase) |
| TraesCS4D01G043500LC.1 | tr\|A0A0B2R0V5\|A0A0B2R0V5_GLYSO | LINE-1 reverse transcriptase like |
| TraesCS4D01G043600LC.1 | AT3G12540.2 | ternary complex factor MIP1 leucine-zipper protein (Protein of unknown function, DUF547) |
| TraesCS4D01G043700LC.1 | tr\|P93369\|P93369_TOBAC | Glutamate decarboxylase |
| TraesCS4D01G043800LC.1 | AT3G19500.3 | basic helix-loop-helix (bHLH) DNA-binding superfamily protein |
| TraesCS4D01G043900LC.1 | sp\|Q03EI3\|G6PI_PEDPA | Glucose-6-phosphate isomerase |
| TraesCS4D01G044000LC.1 | tr\|A0A151TB60\|A0A151TB60_CAJCA | Retrovirus-related Pol polyprotein from transposon TNT 1-94 |
| TraesCS4D01G044100LC.1 | tr\|A0A0D3CKF5\|A0A0D3CKF5_BRAOL | Phospholipid-transporting ATPase |

| | | |
|---|---|---|
| TraesCS4D01G044200LC.1 | tr\|R7WCR4\|R7WCR4_AEGTA | Translational activator GCN1 |
| TraesCS4D01G044300LC.1 | AT5G52790.4 | CBS domain protein with a domain protein (DUF21) |
| TraesCS4D01G044400LC.1 | tr\|G7L3R3\|G7L3R3_MEDTR | Myb-like transcription factor family protein |
| TraesCS4D01G044500LC.1 | sp\|Q1HG44\|DOXA2_HUMAN | Dual oxidase maturation factor 2 |
| TraesCS4D01G044600LC.1 | tr\|M8ASQ8\|M8ASQ8_TRIUA | Mitotic checkpoint protein BUB3 |
| TraesCS4D01G044700LC.1 | tr\|A0A072VQA2\|A0A072VQA2_MEDTR | Cysteine-rich receptor-kinase-like protein |
| TraesCS4D01G044800LC.1 | sp\|Q8DQ18\|GLMU_STRR6 | Bifunctional protein GlmU |
| TraesCS4D01G044900LC.1 | tr\|B9RX67\|B9RX67_RICCO | Kinase, putative |
| TraesCS4D01G045000LC.1 | tr\|A0A1J3HNP1\|A0A1J3HNP1_NOCCA | Glutathione S-transferase T3 |
| TraesCS4D01G045100LC.1 | AT2G02520.1 | RNA-directed DNA polymerase (reverse transcriptase)-related family protein |
| TraesCS4D01G045200LC.1 | tr\|A0A072UHA7\|A0A072UHA7_MEDTR | Endonuclease/exonuclease/phosphatase family protein |
| TraesCS4D01G045300LC.1 | tr\|Q2R1G5\|Q2R1G5_ORYSJ | Transposon protein, putative, Mutator sub-class, expressed |
| TraesCS4D01G045400LC.1 | tr\|A0A078I2C4\|A0A078I2C4_BRANA | DNA helicase |
| TraesCS4D01G045500LC.1 | tr\|A0A1J3HNP1\|A0A1J3HNP1_NOCCA | Glutathione S-transferase T3 |
| TraesCS4D01G045600LC.1 | tr\|A0A151RB35\|A0A151RB35_CAJCA | Retrovirus-related Pol polyprotein from transposon TNT 1-94 |
| TraesCS4D01G045600LC.2 | tr\|A0A151RB35\|A0A151RB35_CAJCA | Retrovirus-related Pol polyprotein from transposon TNT 1-94 |
| TraesCS4D01G045700LC.1 | AT1G23230.2 | mediator of RNA polymerase II transcription subunit |
| TraesCS4D01G045800LC.1 | tr\|F2DNL2\|F2DNL2_HORVV | Xylose isomerase |
| TraesCS4D01G045900LC.1 | sp\|B1XN45\|CLPX_SYNP2 | ATP-dependent Clp protease ATP-binding subunit ClpX |
| TraesCS4D01G046000LC.1 | sp\|P02520\|HSP12_SOYBN | Class I heat shock protein |

| | | |
|---|---|---|
| TraesCS4D01G046100LC.1 | tr\|S4Z1A8\|S4Z1A8_HORVV | 50S ribosomal protein L23, chloroplastic |
| TraesCS4D01G046200LC.1 | AT5G12350.1 | Regulator of chromosome condensation (RCC1) family with FYVE zinc finger domain-containing protein |
| TraesCS4D01G046200LC.2 | AT2G27080.2 | Late embryogenesis abundant (LEA) hydroxyproline-rich glycoprotein family |
| TraesCS4D01G046300LC.1 | tr\|Q53PH7\|Q53PH7_ORYSJ | Retrotransposon protein, putative, unclassified |
| TraesCS4D01G046400LC.1 | tr\|Q2QUY9\|Q2QUY9_ORYSJ | Retrotransposon protein, putative, LINE subclass |
| TraesCS4D01G046500LC.1 | AT2G13510.1 | Ta11-like non-LTR retrotransposon |
| TraesCS4D01G046600LC.1 | tr\|Q2QTW9\|Q2QTW9_ORYSJ | Retrotransposon protein, putative, Ty1-copia subclass |
| TraesCS4D01G046700LC.1 | tr\|A0A151SIM4\|A0A151SIM4_CAJCA | Retrovirus-related Pol polyprotein from transposon TNT 1-94 |
| TraesCS4D01G046800LC.1 | AT3G25270.1 | Ribonuclease H-like superfamily protein |
| TraesCS4D01G046900LC.1 | tr\|M8CRM6\|M8CRM6_AEGTA | Anthocyanin 5-aromatic acyltransferase |
| TraesCS4D01G047000LC.1 | sp\|Q5H4Y7\|ATPD_XANOR | ATP synthase subunit delta |
| TraesCS4D01G047100LC.1 | tr\|Q2R1G5\|Q2R1G5_ORYSJ | Transposon protein, putative, Mutator sub-class, expressed |
| TraesCS4D01G047200LC.1 | tr\|S4TKN2\|S4TKN2_PINPO | Ycf1 |
| TraesCS4D01G047300LC.1 | AT2G01430.2 | homeobox-leucine zipper protein 17 |
| TraesCS4D01G047400LC.1 | tr\|F1DGA5\|F1DGA5_COFAR | Ethylene-responsive transcription factor |
| TraesCS4D01G047500LC.1 | tr\|A0A1E5W1C7\|A0A1E5W1C7_9POAL | F-box/LRR-repeat protein 17 |
| TraesCS4D01G047600LC.1 | sp\|Q4P9Z3\|CLP1_USTMA | mRNA cleavage and polyadenylation factor CLP1 |
| TraesCS4D01G047700LC.1 | tr\|A0A151T2X6\|A0A151T2X6_CAJCA | Retrovirus-related Pol polyprotein from transposon TNT 1-94 |
| TraesCS4D01G047800LC.1 | sp\|Q8TRU6\|RL4_METAC | 50S ribosomal protein L4 |
| TraesCS4D01G047900LC.1 | tr\|A0A151UAS3\|A0A151UAS3_CAJCA | Serine/threonine protein phosphatase 7 long form isogeny |

| | | |
|---|---|---|
| TraesCS4D01G048000LC.1 | tr\|Q53K65\|Q53K65_ORYSJ | Retrotransposon protein, putative, unclassified |
| TraesCS4D01G048100LC.1 | AT3G58040.3 | seven in absentia of Arabidopsis 2 |
| TraesCS4D01G048200LC.1 | sp\|A3QK16\|ERLN2_DANRE | Erlin-2 |
| TraesCS4D01G048300LC.1 | tr\|M8BU25\|M8BU25_AEGTA | Protein FAR1-RELATED SEQUENCE 5 |
| TraesCS4D01G048400LC.1 | tr\|A0A199VPY6\|A0A199VPY6_ANACO | Protein TOPLESS |
| TraesCS4D01G048500LC.1 | tr\|A0A0A9A4K7\|A0A0A9A4K7_ARUDO | Mitochondrial 60S ribosomal protein L6 |
| TraesCS4D01G048600LC.1 | sp\|Q28UY2\|RL11_JANSC | 50S ribosomal protein L11 |
| TraesCS4D01G048700LC.1 | AT3G11960.4 | Cleavage and polyadenylation specificity factor (CPSF) A subunit protein |
| TraesCS4D01G048800LC.1 | tr\|M8CJZ3\|M8CJZ3_AEGTA | Protein FAR1-RELATED SEQUENCE 5 |
| TraesCS4D01G048900LC.1 | tr\|B9HPF8\|B9HPF8_POPTR | Scarecrow transcription factor family protein |
| TraesCS4D01G049000LC.1 | sp\|Q3MFA8\|RS8_ANAVT | 30S ribosomal protein S8 |
| TraesCS4D01G049100LC.1 | AT1G67120.2 | midasin-like protein |
| TraesCS4D01G049200LC.1 | tr\|Q49KC9\|Q49KC9_PEA | Transposase |
| TraesCS4D01G049300LC.1 | tr\|M8BU25\|M8BU25_AEGTA | Protein FAR1-RELATED SEQUENCE 5 |
| TraesCS4D01G049400LC.1 | tr\|M8BGQ5\|M8BGQ5_AEGTA | Protein FAR1-RELATED SEQUENCE 3 |
| TraesCS4D01G049500LC.1 | AT3G12040.1 | DNA-3-methyladenine glycosylase (MAG) |
| TraesCS4D01G049600LC.1 | tr\|S5R947\|S5R947_9ROSA | NAC domain protein |
| TraesCS4D01G049700LC.1 | sp\|Q6F0K3\|SYA_MESFL | Alanine--tRNA ligase |
| TraesCS4D01G049800LC.1 | tr\|K7UGJ4\|K7UGJ4_MAIZE | 50S ribosomal protein L2 |
| TraesCS4D01G049900LC.1 | tr\|A0A1C9II81\|A0A1C9II81_9GENT | Ribosomal protein S19 |

| | | |
|---|---|---|
| TraesCS4D01G050000LC.1 | sp\|Q8ZR29\|ENTH_SALTY | Proofreading thioesterase EntH |
| TraesCS4D01G050100LC.1 | tr\|A0A158N6F3\|A0A158N6F3_CERCN | Photosystem I reaction center subunit IX |
| TraesCS4D01G050200LC.1 | tr\|A0A0F6NMI4\|A0A0F6NMI4_9POAL | ATP synthase subunit beta |
| TraesCS4D01G050300LC.1 | AT3G24255.7 | RNA-directed DNA polymerase (reverse transcriptase)-related family protein |
| TraesCS4D01G050400LC.1 | tr\|A0A1J3FWP0\|A0A1J3FWP0_NOCCA | LINE-1 reverse transcriptase-like protein |
| TraesCS4D01G050500LC.1 | tr\|A0A151RC87\|A0A151RC87_CAJCA | Transposon TX1 uncharacterized |
| TraesCS4D01G050600LC.1 | tr\|Q7XFG3\|Q7XFG3_ORYSJ | Retrotransposon protein, putative, unclassified, expressed |
| TraesCS4D01G050700LC.1 | AT3G24255.5 | RNA-directed DNA polymerase (reverse transcriptase)-related family protein |
| TraesCS4D01G050800LC.1 | tr\|A2Q4L4\|A2Q4L4_MEDTR | RNA-directed DNA polymerase (Reverse transcriptase) |
| TraesCS4D01G050900LC.1 | tr\|A0A199V9F3\|A0A199V9F3_ANACO | LINE-1 reverse transcriptase |
| TraesCS4D01G051000LC.1 | tr\|Q338Z2\|Q338Z2_ORYSJ | Retrotransposon protein, putative, unclassified |
| TraesCS4D01G051100LC.1 | tr\|A0A151SQ16\|A0A151SQ16_CAJCA | Retrovirus-related Pol polyprotein LINE-1 |
| TraesCS4D01G051200LC.1 | tr\|Q5SMW3\|Q5SMW3_ORYSJ | Cyst nematode resistance protein-like |
| TraesCS4D01G051300LC.1 | tr\|A0A1J3HNP1\|A0A1J3HNP1_NOCCA | Glutathione S-transferase T3 |
| TraesCS4D01G051400LC.1 | tr\|Q2QYQ5\|Q2QYQ5_ORYSJ | Retrotransposon protein, putative, unclassified, expressed |
| TraesCS4D01G051500LC.1 | AT5G39410.1 | Saccharopine dehydrogenase |
| TraesCS4D01G051600LC.1 | tr\|B9GHT3\|B9GHT3_POPTR | Plant basic secretory family protein |
| TraesCS4D01G051700LC.1 | tr\|A0A072TJY9\|A0A072TJY9_MEDTR | Endonuclease/exonuclease/phosphatase family protein |
| TraesCS4D01G051800LC.1 | AT2G40030.2 | nuclear RNA polymerase D1B |
| TraesCS4D01G051900LC.1 | tr\|Q53PB1\|Q53PB1_ORYSJ | Serine carboxypeptidase |

| | | |
|---|---|---|
| TraesCS4D01G052000LC.1 | tr\|Q10DG1\|Q10DG1_ORYSJ | Serine carboxypeptidase family protein, expressed |
| TraesCS4D01G052100LC.1 | tr\|Q2QP96\|Q2QP96_ORYSJ | Transposon protein, putative, CACTA, En/Spm sub-class |
| TraesCS4D01G052200LC.1 | tr\|Q10HY9\|Q10HY9_ORYSJ | Retrotransposon protein, putative, unclassified |
| TraesCS4D01G052300LC.1 | AT5G45260.3 | Disease resistance protein (TIR-NBS-LRR class) |
| TraesCS4D01G052400LC.1 | tr\|U5GRI5\|U5GRI5_POPTR | Oxysterol-binding family protein |
| TraesCS4D01G052500LC.1 | sp\|Q145F1\|DNAK_BURXL | Chaperone protein DnaK |
| TraesCS4D01G052600LC.1 | tr\|A0A1E5WHY8\|A0A1E5WHY8_9POAL | Linolenate hydroperoxide lyase, chloroplastic |
| TraesCS4D01G052700LC.1 | tr\|A0A072VDU8\|A0A072VDU8_MEDTR | Endo-1,4-beta-xylanase A-like protein |
| TraesCS4D01G052800LC.1 | tr\|A0A1D5X4U4\|A0A1D5X4U4_WHEAT | Endonuclease III homolog |
| TraesCS4D01G052900LC.1 | AT1G63770.7 | Peptidase M1 family protein |
| TraesCS4D01G053000LC.1 | sp\|A6T221\|PANB_JANMA | 3-methyl-2-oxobutanoate hydroxymethyltransferase |
| TraesCS4D01G053100LC.1 | AT4G29090.1 | Ribonuclease H-like superfamily protein |
| TraesCS4D01G053200LC.1 | tr\|W9RW78\|W9RW78_9ROSA | F-box protein |
| TraesCS4D01G053300LC.1 | tr\|Q5D214\|Q5D214_ORYSJ | Cathepsin B-like cysteine protease |
| TraesCS4D01G053400LC.1 | tr\|S5R947\|S5R947_9ROSA | NAC domain protein |
| TraesCS4D01G053500LC.1 | tr\|Q5D214\|Q5D214_ORYSJ | Cathepsin B-like cysteine protease |
| TraesCS4D01G053600LC.1 | tr\|Q2QZV1\|Q2QZV1_ORYSJ | Retrotransposon protein, putative, unclassified |
| TraesCS4D01G053700LC.1 | tr\|A0A199V9F3\|A0A199V9F3_ANACO | LINE-1 reverse transcriptase |
| TraesCS4D01G053800LC.1 | tr\|A0A199VZT9\|A0A199VZT9_ANACO | Peptide-N4-(N-acetyl-beta-glucosaminyl)asparagine amidase A |
| TraesCS4D01G053900LC.1 | tr\|Q94LG5\|Q94LG5_ORYSJ | Transposon protein, putative, CACTA, En/Spm sub-class |

| | | |
|---|---|---|
| TraesCS4D01G054000LC.1 | tr\|Q2R455\|Q2R455_ORYSJ | Transposon protein, putative, CACTA, En/Spm sub-class |
| TraesCS4D01G054100LC.1 | tr\|A0A061G9M1\|A0A061G9M1_THECC | Non-LTR retroelement reverse transcriptase |
| TraesCS4D01G054200LC.1 | AT4G29090.1 | Ribonuclease H-like superfamily protein |
| TraesCS4D01G054300LC.1 | sp\|Q07889\|SOS1_HUMAN | Son of sevenless homolog 1 |
| TraesCS4D01G054400LC.1 | AT1G58410.4 | Disease resistance protein (CC-NBS-LRR class) family |
| TraesCS4D01G054500LC.1 | AT4G18020.9 | CheY-like two-component responsive regulator family protein |
| TraesCS4D01G054600LC.1 | sp\|Q9NXW9\|ALKB4_HUMAN | Alpha-ketoglutarate-dependent dioxygenase alkB homolog 4 |
| TraesCS4D01G054700LC.1 | | Unknown protein |
| TraesCS4D01G054800LC.1 | tr\|G4XYW2\|G4XYW2_BRAJU | Orf115b |
| TraesCS4D01G054900LC.1 | tr\|Q2QVI6\|Q2QVI6_ORYSJ | Transposon protein, putative, Pong sub-class |
| TraesCS4D01G055000LC.1 | sp\|O97176\|ESM1_DROME | Enhancer of split M1 protein |
| TraesCS4D01G055100LC.1 | tr\|M8C787\|M8C787_AEGTA | B3 domain-containing protein |
| TraesCS4D01G055200LC.1 | tr\|Q33AJ4\|Q33AJ4_ORYSJ | Retrotransposon protein, putative, unclassified |
| TraesCS4D01G055300LC.1 | tr\|R7WBG9\|R7WBG9_AEGTA | 50S ribosomal protein L7/L12 |
| TraesCS4D01G055400LC.1 | tr\|A0A0B0MS63\|A0A0B0MS63_GOSAR | Glyceraldehyde-3-phosphate dehydrogenase |
| TraesCS4D01G055500LC.1 | tr\|Q7XD58\|Q7XD58_ORYSJ | Ulp1 protease family, C-terminal catalytic domain containing protein |
| TraesCS4D01G055600LC.1 | tr\|E9KJ97\|E9KJ97_9ORYZ | NAD(P)H-quinone oxidoreductase subunit J, chloroplastic |
| TraesCS4D01G055700LC.1 | sp\|O01501\|CCNE_CAEEL | G1/S-specific cyclin-E |
| TraesCS4D01G055800LC.1 | tr\|A0A151QRU6\|A0A151QRU6_CAJCA | Retrovirus-related Pol polyprotein from transposon TNT 1-94 |
| TraesCS4D01G055900LC.1 | sp\|P03355\|POL_MLVMS | Gag-Pol polyprotein |

| | | |
|---|---|---|
| TraesCS4D01G056000LC.1 | sp\|P10272\|POL_BAEVM | Pol polyprotein |
| TraesCS4D01G056100LC.1 | tr\|S5R952\|S5R952_9ROSA | NAC domain protein |
| TraesCS4D01G056200LC.1 | tr\|A0A151UBY9\|A0A151UBY9_CAJCA | Retrovirus-related Pol polyprotein from transposon TNT 1-94 |
| TraesCS4D01G056300LC.1 | AT4G09660.1 | zinc finger MYM-type-like protein |
| TraesCS4D01G056400LC.1 | tr\|A0A151S3H8\|A0A151S3H8_CAJCA | Transposon Ty3-G Gag-Pol polyprotein |
| TraesCS4D01G056500LC.1 | tr\|Q0WZC2\|Q0WZC2_WHEAT | RNA ligase isoform 1 |
| TraesCS4D01G056600LC.1 | tr\|Q0WZC1\|Q0WZC1_WHEAT | RNA ligase isoform 2 |
| TraesCS4D01G056700LC.1 | AT5G63930.1 | Leucine-rich repeat protein kinase family protein |
| TraesCS4D01G056800LC.1 | sp\|Q30T61\|ILVC_SULDN | Ketol-acid reductoisomerase (NADP(+)) |
| TraesCS4D01G056900LC.1 | tr\|Q6US98\|Q6US98_9ASPA | Transposase |
| TraesCS4D01G057000LC.1 | tr\|A0A072VJB5\|A0A072VJB5_MEDTR | Myb/SANT-like DNA-binding domain protein |
| TraesCS4D01G057100LC.1 | tr\|G7KQY4\|G7KQY4_MEDTR | Beta-fructofuranosidase, insoluble protein |
| TraesCS4D01G057200LC.1 | tr\|Q60DJ3\|Q60DJ3_ORYSJ | Transposon protein, putative, mutator sub-class |
| TraesCS4D01G057300LC.1 | AT5G61020.2 | evolutionarily conserved C-terminal region 3 |
| TraesCS4D01G057400LC.1 | AT4G01020.1 | helicase domain-containing protein / IBR domain-containing protein / zinc finger protein-like protein |
| TraesCS4D01G057500LC.1 | tr\|M7YSL0\|M7YSL0_TRIUA | Retrovirus-related Pol polyprotein from transposon TNT 1-94 |
| TraesCS4D01G057600LC.1 | tr\|Q2HS45\|Q2HS45_MEDTR | RNA-directed DNA polymerase (Reverse transcriptase) |
| TraesCS4D01G057700LC.1 | sp\|Q7MZ25\|SYV_PHOLL | Valine--tRNA ligase |
| TraesCS4D01G057800LC.1 | sp\|B1KD83\|GLAA_SHEWM | Alpha-1,3-galactosidase A |
| TraesCS4D01G057900LC.1 | sp\|B7GTU6\|GATA_BIFLS | Glutamyl-tRNA(Gln) amidotransferase subunit A |

| | | |
|---|---|---|
| TraesCS4D01G058000LC.1 | tr\|A0A061DTI0\|A0A061DTI0_THECC | ERD (Early-responsive to dehydration stress) family protein |
| TraesCS4D01G058100LC.1 | AT4G24230.6 | acyl-CoA-binding domain 3 |
| TraesCS4D01G058200LC.1 | AT3G19820.3 | cell elongation protein / DWARF1 / DIMINUTO (DIM) |
| TraesCS4D01G058300LC.1 | AT4G04650.1 | RNA-directed DNA polymerase (reverse transcriptase)-related family protein |
| TraesCS4D01G058400LC.1 | AT2G34660.3 | multidrug resistance-associated protein 2 |
| TraesCS4D01G058500LC.1 | tr\|A0A1D1XPP2\|A0A1D1XPP2_9ARAE | Protein EMSY |
| TraesCS4D01G058600LC.1 | AT1G07670.2 | endomembrane-type CA-ATPase 4 |
| TraesCS4D01G058700LC.1 | tr\|A0A1D6KVJ2\|A0A1D6KVJ2_MAIZE | Phospholipid-transporting ATPase |
| TraesCS4D01G058800LC.1 | AT2G04420.1 | Polynucleotidyl transferase, ribonuclease H-like superfamily protein |
| TraesCS4D01G058900LC.1 | AT2G28290.6 | P-loop containing nucleoside triphosphate hydrolases superfamily protein |
| TraesCS4D01G059000LC.1 | tr\|A0A1J3HNP1\|A0A1J3HNP1_NOCCA | Glutathione S-transferase T3 |
| TraesCS4D01G059100LC.1 | AT4G27370.4 | P-loop containing nucleoside triphosphate hydrolases superfamily protein |
| TraesCS4D01G059200LC.1 | tr\|Q5ZCW4\|Q5ZCW4_ORYSJ | F-box domain containing protein-like |
| TraesCS4D01G059300LC.1 | tr\|A0A1D1Y1D5\|A0A1D1Y1D5_9ARAE | Retrovirus-related Pol polyprotein from transposon TNT 1-94 |
| TraesCS4D01G059400LC.1 | sp\|A4IQU0\|MNTR_GEOTN | Transcriptional regulator MntR |
| TraesCS4D01G059500LC.1 | sp\|Q2KI97\|GPR84_BOVIN | G-protein coupled receptor 84 |
| TraesCS4D01G059600LC.1 | tr\|A0A061GX88\|A0A061GX88_THECC | Fasciclin-like arabinogalactan family protein |
| TraesCS4D01G059700LC.1 | AT4G33110.2 | S-adenosyl-L-methionine-dependent methyltransferases superfamily protein |
| TraesCS4D01G059800LC.1 | sp\|Q92796\|DLG3_HUMAN | Disks large homolog 3 |
| TraesCS4D01G059900LC.1 | tr\|Q2QPB0\|Q2QPB0_ORYSJ | Transposon protein, putative, Mutator sub-class |

| | | |
|---|---|---|
| TraesCS4D01G060000LC.1 | tr\|A0A0K0XR78\|A0A0K0XR78_TOBAC | Serine/threonine-protein kinase |
| TraesCS4D01G060100LC.1 | tr\|A0A0K9PGX3\|A0A0K9PGX3_ZOSMR | DNA-(Apurinic or apyrimidinic site) lyase |
| TraesCS4D01G060200LC.1 | sp\|Q8BJW7\|EME1_MOUSE | Crossover junction endonuclease EME1 |
| TraesCS4D01G060300LC.1 | tr\|A0A199W1B4\|A0A199W1B4_ANACO | LINE-1 retrotransposable element ORF2 protein |
| TraesCS4D01G060400LC.1 | sp\|Q8FXT2\|IF2_BRUSU | Translation initiation factor IF-2 |
| TraesCS4D01G060500LC.1 | tr\|A0A072VJB5\|A0A072VJB5_MEDTR | Myb/SANT-like DNA-binding domain protein |
| TraesCS4D01G060600LC.1 | sp\|Q8FM78\|DNAK_COREF | Chaperone protein DnaK |
| TraesCS4D01G060700LC.1 | tr\|W9S8P3\|W9S8P3_9ROSA | Ribosomal RNA large subunit methyltransferase N 1 |
| TraesCS4D01G060800LC.1 | tr\|A0A087HMA2\|A0A087HMA2_ARAAL | Phytochrome-interacting factor |
| TraesCS4D01G060900LC.1 | tr\|Q2QYQ5\|Q2QYQ5_ORYSJ | Retrotransposon protein, putative, unclassified, expressed |
| TraesCS4D01G061000LC.1 | tr\|A0A1J3FWP0\|A0A1J3FWP0_NOCCA | LINE-1 reverse transcriptase-like protein |
| TraesCS4D01G061100LC.1 | tr\|Q7XFG3\|Q7XFG3_ORYSJ | Retrotransposon protein, putative, unclassified, expressed |
| TraesCS4D01G061200LC.1 | tr\|Q5SMW3\|Q5SMW3_ORYSJ | Cyst nematode resistance protein-like |
| TraesCS4D01G061300LC.1 | tr\|Q2QZV1\|Q2QZV1_ORYSJ | Retrotransposon protein, putative, unclassified |

Note: Genes in red font indicate those directly related to heat toleranc

## Supplementary Table S3. Comparison of identified QTL with previously reported ones

| | QTLs in this study | | | QTLs in previous study | | | | | | Distance |
|---|---|---|---|---|---|---|---|---|---|---|
| **Chr** | **Major QTLs** | **Position** | **Other reported QTLs** | **Flanking Markers** | **Position** | **Traits** | **Stress** | **No. of QTLs** | **Reference** | **Physical Distance** |
| 2D | *QRl.uwa.2D* | 34894665-Xcdo1379 | *MQTL2D.3* | wsnp_Ex_c29666_38670435 – Tdurum_contig5311_112 | 62288723–75506740 | GY-all; GN; HSI(TGW) | HS | 5 | Liu *et al.*, 2020 | 27394058 |
| | | | *Q.Irwc.cgb-2D* | P3176.1–P1123.1 | | Index of leaves relative water content | HS | | Li *et al.*, 2013 | |
| | | | *QTdl.tam09-2D* | Xcfd56 | | Temperature depression of flag leaf | HS | | Mondal *et al.*, 2015 | |
| 3D | *QRlhti.uwa.3D* | 39413890-XksuA6 | *MQTL3D.1* | IAAV2729–GENE-1919_120 | 51042786–86353210 | GY-DS;GN-DH;HI-DS | DS + HS | 3 | Liu *et al.*, 2020 | 11628896 |
| | | | *Q.Yld.aww-3D* | Xcfd34–Xwmc0533 | | Grain yield | | | Bennett *et al.*, 2012 | |
| | | | *QWax.tam09-3D* | Xgwm191 | | Flag leaf cuticular waxes | HS | | Mondal *et al.*, 2015 | |
| | | | *Q.Itgw.cgb-3D* | Xgwm456–Xgdm8 | | Index of thousand-grain weight | HS | | Li *et al.*, 2013 | |
| | | | *QGwt.crc3D* | tplb0029j24_2118-wsnp_Ex_rep_c101732_87042471 | | Grain weight | | | | |
| 4D | *QSlhti.uwa.4D* | 3327302-37048569 | *MQTL4D.3* | BobWhite_c4264_200 – RAC875_c40619_130 | 121181572–348798389 | HI-all; SN-all; GY-HS | HS | 3 | Liu *et al.*, 2020 | 84133003 |
| | *QRlhti.uwa.4D* | 3327302-37048569 | *Q.Yld.aww-4D* | Xwmc0457–Xbarc0288 | Xwmc0457–121414029 | Grain yield | | | Bennett *et al.*, 2012 | 84365460 |
| | *QWlhti.uwa.4D* | 3327302-37048569 | *MQTL35* | | | HD, 2CI D, 4Col, HI, KN, Mat, Photo, Sm2, SG, TW, 3TKW, Yld | DS + HS | | Andrea Acuña-Galindo *et al.*, 2015 | |
| | *QSl.uwa.4D* | 3327302-37048569 | *MQTL36* | | | Col, Hgt, Yld | HS | | | |
| | *QRl.uwa.4D* | 3327302-37048569 | | | | | | | | |
| | *QWl.uwa.4D* | 3327302-37048569 | | | | | | | | |
| 5A | *QWl.uwa.5A* | 560132505-Xbod183 | *MQTL5A.3* | GENE-3493_612 – wsnp_Ex_c19647_28632894 | 461519115–470033346 | GY-all;GN-DH | DS + HS | 2 | Liu *et al.*, 2020 | 90099159 |
| | | | *MQTL5A.5* | Kukri_c40919_372 – BS00109052_51 | 445287898–504759564 | GY-all;GN-DH | DS + HS | 3 | Liu *et al.*, 2020 | 55372941 |
| | | | *MQTL5A.6* | Kukri_rep_c107435_940 – BobWhite_c5917_529 | 46621851–47459518 | GY-DS;GN-HS;GN-DH;GY-DH | HS,DS + HS | 4 | Liu *et al.*, 2020 | 512672987 |
| | | | *Xgwm293-linked QTL* | Xgwm293 | | GFD | HS | | Yang *et al.*, 2002 | |

| | | | | | | |
|---|---|---|---|---|---|---|
| *QTds.tam09-5A* | Xgwm293 | 105903106-105902907 | Temperature depression of main spike | HS | Mondal *et al*., 2015 | 454229598 |
| *QWax.tam09-5A* | Xgwm205 | | Flag leaf cuticular waxes | HS | Mondal *et al*., 2015 | |
| *QWax.tam08-5A* | Xwmc713 | | Flag leaf cuticular waxes | HS | Mondal *et al*., 2015 | |
| *QTdl.tam09-5A* | Xbarc141 | 469408810-469408446 | Temperature depression of flag leaf | HS | Mondal *et al*., 2015 | 90723695 |

*GY* grain yield per unit (square meter or plant), *TGW* thousand-grain weight or grain weight per grain, *HSI* heat susceptibility index, *DS* drought stress, *GFD* grain filling duration; *HS* heat stress, *NS* non-stress, *all* all environments with no specific stress description
*CI D*, carbon isotope discrimination; *Col*, coleoptile vigor; *HD*, Heading/Anthesis; *hgt*, height; *HI*, harvest index; *KN*, kernel number; *Mat*, maturity; *Photo*, photosynthesis; *SG*, stay-green; sm2, spike density; *TW*, test weight; *Yld*, yield.

**Supplementary Table S4. Reported Yield-related genomic regions within the chromosome 4D hotspot**

| QTL or MTA | Traits | Flanking markers | Physical positionon | Reference |
|---|---|---|---|---|
| **MQTL** | | | | |
| MQTL-4D-1 | GW, GFR, GN, GY | Kukri_rep_c106474_293–Excalibur_c91022_193 | 6.02–7.65 Mbp | Yang et al., 2021 |
| MQTL-4D-2 | GW, GY, SLN, TN, GN, SL, HI | | 1076436–1672793 | Yang et al., 2021 |
| MQTL4D.1 | SN-all; TGW-all | Rht2–GENE-3024_59 | 19189381–19301407 | Liu et al., 2020 |
| MQTL4D.2 | GN-all; TGW-all;SN-all | Xwmc48–BS00094770_51 | 335782060–361802106 | Liu et al., 2020 |
| MQTL4D.3 | HI-all; SN-all;GY-HS | BobWhite_c4264_200–RAC875_c40619_130 | 121181572–348798389 | Liu et al., 2020 |
| MQTL4D.4 | GY-all; HI-all | BS00065818_51–Xcfd23 | 126644126–281881023 | Liu et al., 2020 |
| **Original QTL** | | | | |
| 4DQTL1 | GN-all | Xwmc48 - Xcfd23 | | McIntyre et al. 2010 |
| 4DQTL2 | HI-all | Xwmc48 - Xcfd23 | | McIntyre et al. 2010 |
| 4DQTL3 | GW-all | Xwmc48 - Xcfd23 | | McIntyre et al. 2010 |
| 4DQTL4 | SN-all | Xwmc48 - Xcfd23 | | McIntyre et al. 2010 |
| 4DQTL5 | SN | Rht2 | | Guan et al. 2018 |
| 4DQTL6 | SN | wsnp_Ex_c13859_21713144 | | Guan et al. 2018 |
| 4DQTL7 | GW | Rht2 | | Guan et al. 2018 |
| 4DQTL8 | GW | Xbarc105 | | Guan et al. 2018 |
| 4DQTL9 | TGW | Kukri_c3489_714 | | Guan et al. 2018 |

| | | | | |
|---|---|---|---|---|
| 4DQTL10 | GY-HS | WMC0457-BARC0288 | | Bennett et al. 2012 |
| 4DQTL11 | GY | psp3103 | | Czyczylo-Mysza et al. 2013 |
| 4DQTL12 | TGW-all | wmc617c - wMAS000002 | | Cabral et al. 2018 |
| 4DQTL13 | TGW-all | X1004846–X1161775 | | Tura et al. 2020 |
| **MTA** | | | | |
| 4DMTA1 | YLD | | 2985475 | Juliana et al. 2019 |
| 4DMTA2 | GN | | 3983968 | Li et al. 2019 |
| 4DMTA3 | GN | | 474583434 | Li et al. 2019 |
| 4DMTA4 | TGW | | 509426797-509429279 | Bhatta et al. 2018 |

CI, confidence interval; SN, spike number per unit (square meter or plant); GN, grain number per unit (square meter, spike or plant); GW, Grain weight; GY, grain yield per unit (square meter or plant); TN, Tiller number; SL, Spike length; SLN, Spikelet number; TGW, thousand-grain weight or grain weight per grain; GFR; grain filling rate; HI, harvest index; HS, heat stress; all, all environments with no specific stress description.

The reported genomic region overlap or within the QTL hotspot in this study was marked in red.

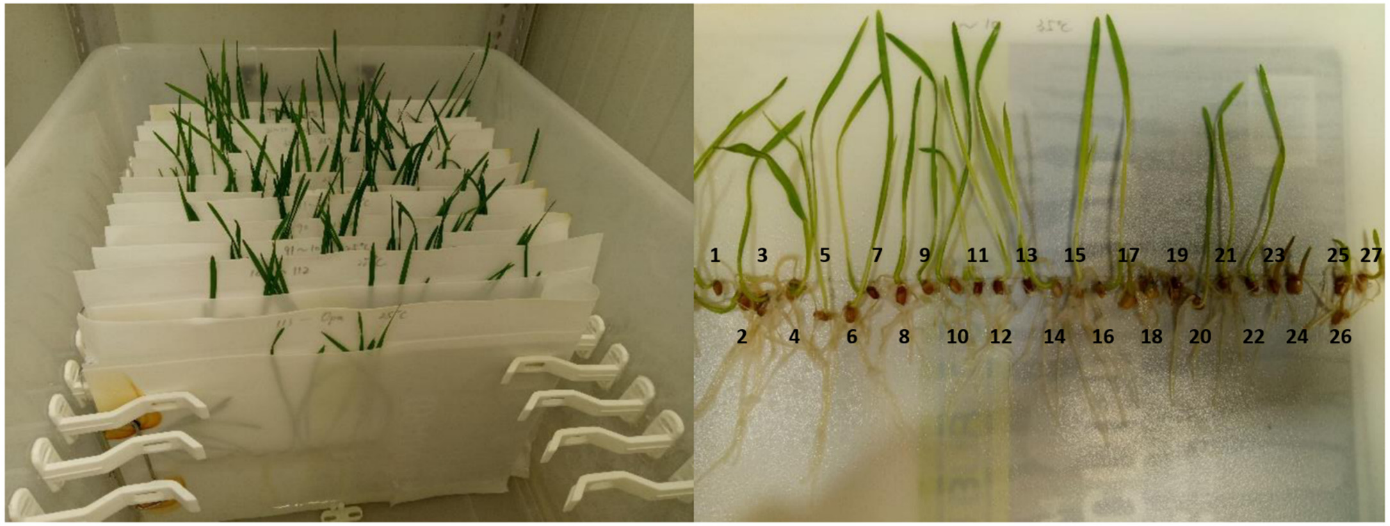


Figure S1. Illustration of the hydroponic growth system (modified from Fig. S1 in Lu et al., 2022). The folders were marked in sequence. The holder system with clip folders inside is shown on the left, and an example of plant growth inside the folder is shown on the right of the figure. Only 27 seeds for 9 lines on one clip folder under one condition was shown in the figure as a representative. In this study, a total of 339 individual plants (two parents and 111 ITMI lines with three replications for each genotype per treatment) were grown for data collection.
